# JAK-STAT signaling maintains homeostasis in T cells and macrophages

**DOI:** 10.1038/s41590-024-01804-1

**Published:** 2024-04-24

**Authors:** Nikolaus Fortelny, Matthias Farlik, Victoria Fife, Anna-Dorothea Gorki, Caroline Lassnig, Barbara Maurer, Katrin Meissl, Marlies Dolezal, Laura Boccuni, Aarathy Ravi Sundar Jose Geetha, Mojoyinola Joanna Akagha, Anzhelika Karjalainen, Stephen Shoebridge, Asma Farhat, Ulrike Mann, Rohit Jain, Shweta Tikoo, Nina Zila, Wolfgang Esser-Skala, Thomas Krausgruber, Katarzyna Sitnik, Thomas Penz, Anastasiya Hladik, Tobias Suske, Sophie Zahalka, Martin Senekowitsch, Daniele Barreca, Florian Halbritter, Sabine Macho-Maschler, Wolfgang Weninger, Heidi A. Neubauer, Richard Moriggl, Sylvia Knapp, Veronika Sexl, Birgit Strobl, Thomas Decker, Mathias Müller, Christoph Bock

**Affiliations:** 1 CeMM Research Center for Molecular Medicine of the Austrian Academy of Sciences, Vienna, Austria; 2 Center for Tumor Biology and Immunology, Department of Biosciences and Medical Biology, Paris-Lodron University Salzburg, Salzburg, Austria; 3 Department of Dermatology, Medical University of Vienna, Vienna, Austria; 4 Research Division of Infection Biology, Department of Medicine I, Medical University of Vienna, Vienna, Austria; 5 Animal Breeding and Genetics and VetBiomodels, Department of Biological Sciences and Pathobiology, University of Veterinary Medicine, Vienna, Austria; 6 Pharmacology and Toxicology, Department of Biological Sciences and Pathobiology, University of Veterinary Medicine, Vienna, Austria; 7 Platform for Bioinformatics and Biostatistics, Department of Biological Sciences and Pathobiology, University of Veterinary Medicine, Vienna, Austria; 8 Max Perutz Labs, University of Vienna, Vienna, Austria; 9 Institute of Artificial Intelligence, Center for Medical Data Science, Medical University of Vienna, Vienna, Austria; 10 Present Address: University of Innsbruck, Innsbruck, Austria

**Keywords:** Gene regulation in immune cells, Gene expression analysis, Epigenetics analysis

## Abstract

Immune cells need to sustain a state of constant alertness over a lifetime. Yet, little is known about the regulatory processes that control the fluent and fragile balance that is called homeostasis. Here we demonstrate that JAK-STAT signaling, beyond its role in immune responses, is a major regulator of immune cell homeostasis. We investigated JAK-STAT-mediated transcription and chromatin accessibility across 12 mouse models, including knockouts of all STAT transcription factors and of the TYK2 kinase. Baseline JAK-STAT signaling was detected in CD8^+^ T cells and macrophages of unperturbed mice—but abrogated in the knockouts and in unstimulated immune cells deprived of their normal tissue context. We observed diverse gene-regulatory programs, including effects of STAT2 and IRF9 that were independent of STAT1. In summary, our large-scale dataset and integrative analysis of JAK-STAT mutant and wild-type mice uncovered a crucial role of JAK-STAT signaling in unstimulated immune cells, where it contributes to a poised epigenetic and transcriptional state and helps prepare these cells for rapid response to immune stimuli.

## Abstract (web summary)

Bock and colleagues perform integrative analysis of JAK-STAT mutant mice and find JAK-STAT signaling regulates CD8^+^ T cell and macrophage homeostasis by contributing to a poised epigenetic and transcription-regulatory state, preparing cells to rapidly respond to stimuli.

## Main

The concept of cellular homeostasis refers to the ability of cells to actively maintain a viable and functional state over time. For immune cells, which need to respond rapidly to potential threats such as infection or tissue damage^1–3^, this includes maintaining constant alertness under homeostatic conditions (that is, in the absence of stimuli that can trigger an active immune response). Importantly, mammalian immune cells do not use a simple on–off switch between homeostatic maintenance and immune activation. Rather, they appear to implement gradual regulatory processes with baseline activity under homeostatic conditions and rapid upregulation of key immune signaling pathways when the cells encounter pathogens or other immune stimuli^4,5^.

Immune cells employ signaling pathways to transmit cell-extrinsic immune stimuli to the nucleus, where they trigger specific transcriptional programs associated with acute immune responses^6,7^. These pathways usually comprise cell surface receptors, signal transducers such as kinases and transcription factors that regulate their target gene sets^8,9^. JAK-STAT signaling is a prototypical example of an immune response pathway^10–12^. Cytokine receptors with associated JAK-family kinases phosphorylate STAT-family transcription factors, which transition to the nucleus and regulate specific target genes, thus enabling rapid cellular information processing.

In mouse and human, JAK-STAT signaling comprises four different JAKs and seven different STATs, which control a broad range of biological functions relevant to the response to immune stimuli^13,14^. STAT proteins bind two types of promoter sequences: (1) the GAS element is bound by all STAT homodimers and heterodimers except STAT1-STAT2; (2) the ISRE element is bound by the interferon (IFN)-activated ISGF3 complex, which consists of a STAT1-STAT2 heterodimer complexed with the IRF9 transcription factor^15–17^.

Under homeostatic conditions, one would expect IFN signaling and ISGF3 activity to be silenced and stably repressed, given that they target many proinflammatory genes whose inappropriate activation is likely to harm the host through excess inflammation and ensuing tissue damage. Nevertheless, previous studies found low-level expression of STAT1 and STAT2 target genes in the absence of exogenous stimuli^18–21^. While this observation suggests that JAK-STAT signaling may retain baseline activity under homeostatic conditions, the means and purposes of JAK-STAT signaling under homeostatic conditions remain poorly understood.

**Extended Data Fig. 1:**
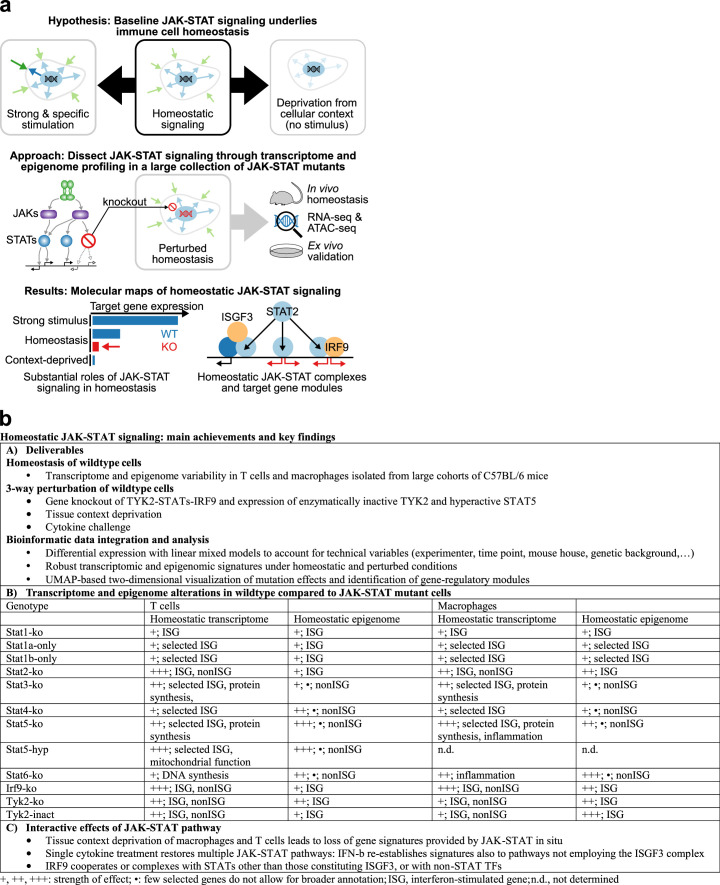
Study overview. Illustration of the study design and key results as a graphical abstract (**a**) and a summary table of transcriptome and epigenome alterations in JAK-STAT mutant cells (**b**). We hypothesized that baseline JAK-STAT signaling underlies immune cell homeostasis, beyond its well-established role during immune stimulation. We analyzed homeostatic JAK-STAT signaling through transcriptome and epigenome profiling in a large collection of JAK-STAT mutants and validated our results by depriving cells from their cellular context in ex vivo culture. Our molecular maps of homeostatic JAK-STAT signaling reveal substantial roles of JAK-STAT signaling in specific homeostatic JAK-STAT complexes and regulated gene modules.

Here we pursue the hypothesis that JAK-STAT signaling, in addition to its established role in active immune responses, constitutes a major regulator of immune cell homeostasis (for the purpose of this study, we operationally defined homeostasis as the unperturbed state of immune cells obtained from wild-type laboratory mice that live under specific-pathogen-free conditions in a normally clean animal house). We obtained CD8^+^ T cells and macrophages from 12 JAK-STAT mutant mouse models under homeostatic conditions and subjected these immune cells to transcription profiling and chromatin accessibility mapping (Extended Data Fig. 1).

Our analysis uncovered genes and gene-regulatory modules that are controlled by JAK-STAT pathway members under homeostatic conditions. We observed widespread baseline activity of JAK-STAT signaling, with STAT2 and IRF9 as the most important regulators. STAT1 knockout had less pronounced effects, despite its key role in the IFN-stimulated gene factor (ISGF3) complex. We functionally assessed the homeostatic roles of JAK-STAT signaling by removing wild-type cells from their in vivo tissue context, which resulted in transcriptional changes that mimicked those observed in JAK-STAT mutants. This context deprivation phenotype was partially rescued by type I IFN stimulation of wild-type and JAK-STAT mutant cells. In summary, our study establishes baseline JAK-STAT activity as a key mediator of homeostasis in unstimulated immune cells.

## Results

### Transcription regulation by JAK-STAT in immune homeostasis

**Fig. 1:**
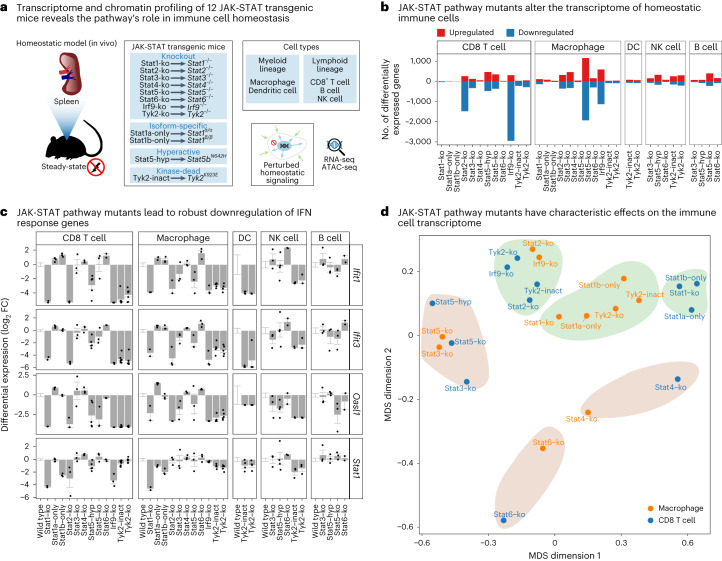
Transcriptome effects of JAK-STAT mutants in homeostasis. **a**, Outline of the experimental approach for dissecting the gene-regulatory landscape of JAK-STAT signaling under homeostatic conditions. **b**, Bar plots showing the number of differentially expressed genes (at a 5% FDR cutoff and FC greater than 2) between JAK-STAT mutant and wild-type mice in five immune cell types. **c**, Gene expression for selected IFN response genes in immune cells from JAK-STAT mutant and wild-type mice. Bar plots display the mean and standard error. **d**, Similarity of transcriptional effects of JAK-STAT mutant mice in T cells and macrophages, based on multi-dimensional scaling (MDS) of Spearman correlation coefficients among log_2_FCs compared with wild-type mice. Results for all cell types are shown in Extended Data Fig. 2d,e. FDR, false discovery rate; log_2_FC, log_2_ fold change.

JAK-STAT signaling is an important regulatory pathway and a plausible candidate for controlling immune cell homeostasis. Building upon decades of research on JAK-STAT signaling in immunology and development^22,23^, recent studies utilized RNA sequencing (RNA-seq) and epigenome profiling to investigate JAK-STAT signaling in response to acute immune stimuli^24–33^. However, a systematic analysis of JAK-STAT in homeostasis has been lacking. We thus mapped and analyzed the transcriptomes and epigenomes of homeostatic immune cells for 12 JAK-STAT mouse models, including knockouts and function-altering mutants (Fig. 1a). We focused our analyses on sort-purified CD8^+^ T cells and macrophages from spleen, thus covering both the lymphoid and myeloid lineage with cell types that show robust expression of JAK-STAT proteins. In addition to our main focus on CD8^+^ T cells and F4/80^+^ macrophages, we also investigated MHCII^+^ CD11c^+^ dendritic cells, NK1.1^+^ natural killer (NK) cells and CD19^+^ B cells for some of the mouse models (Supplementary Fig. 1).

We included knockouts of all STATs (STAT1, STAT2, STAT3, STAT4, STAT5a/b, STAT6) as the pathway’s regulators of transcription and chromatin. Because knockouts of STAT3 and STAT5 are embryonically lethal^34–37^, we studied these two transcription factors using conditional knockouts in hematopoietic cells (Vav-iCre). For in-depth analysis of STAT1, we further included two isoform-specific mouse models: STAT1 beta-only mutant (Stat1b-only, where only Stat1β is expressed) and STAT1 alpha-only mutant (Stat1a-only, where only STAT1α is expressed). We also included knockout mice for the STAT cofactor IRF9, and mice with the hyperactivating, oncogenic STAT5B^N642H^ (Stat5b-hyp) mutation. Finally, we included Janus kinase TYK2 knockout mice and kinase-dead TYK2^K923E^ mutant mice (Tyk2-inact), to assess kinase-independent effects. We did not include knockouts of the Janus kinases JAK1, JAK2 and JAK3 because these are perinatally or embryonically lethal (JAK1, JAK2) and interfere with normal hematopoiesis (JAK1, JAK2, JAK3)^14,38^.

**Extended Data Fig. 2:**
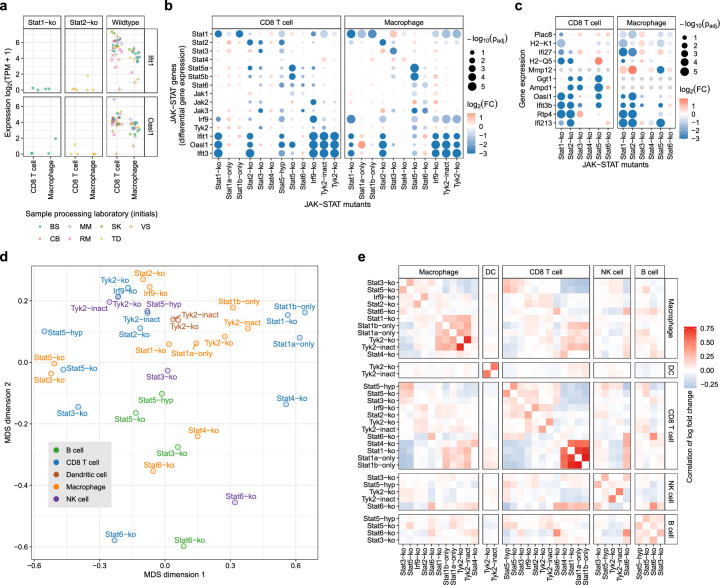
Transcriptome effects of JAK-STAT mutants in five immune cell types. (**a**) Expression levels of two classical IFN target genes across cell types, mutants and sample processing laboratories. Downregulation of genes upon JAK-STAT mutants (differences to wildtype) strongly exceeded experimental variability across laboratories (variability within wildtype samples, indicated by the shape and size of points). (**b**) Effects of mutants on gene expression levels of JAK-STAT genes (two-sided linear mixed models, corrected for multiple comparisons). (**c**) Analysis of a pan-STAT signature (two-sided linear mixed models, corrected for multiple comparisons), showing only genes affected by multiple STAT mutants are shown. No gene was affected by all STAT mutants. (**d-e**) Transcriptional effects of JAK-STAT-mutant mice in all cell types, visualized by multi-dimensional scaling (MDS) (**d**) of Spearman correlation coefficients among log_2_FCs compared to wildtype mice (**e**). FC: fold change; padj: adjusted p-value.

In total, we obtained 469 high-quality transcriptomes by RNA-seq and 496 high-quality epigenome profiles with the assay for transposase-accessible chromatin using sequencing (ATAC-seq) (Supplementary Table 1). All samples were processed according to well-defined standard operating procedures to enhance consistency across six laboratories and three mouse facilities in our consortium. We always processed wild-type mice along with the JAK-STAT mice to control for batch effects (such as mouse facility, processing date or experimenter). We extensively validated the quality, sensitivity and robustness of our dataset (Extended Data Fig. 2a, Supplementary Figs. 2–4 and Supplementary Note). All data are available for download and for interactive browsing as UCSC Genome Browser tracks (http://jakstat.bocklab.org).

Our analysis uncovered characteristic regulatory roles of all investigated JAK-STAT proteins (Fig. 1b and Supplementary Table 2). Knockout of STAT2, STAT3, STAT5 and IRF9 had the strongest transcriptional consequences (Fig. 1b). Knockout of STAT1 or one of its isoforms had smaller effects, despite its prominent role in the ISGF3 complex. For IFN-stimulated genes (ISGs) as prototypical targets of JAK-STAT signaling, we observed marked downregulation in knockouts of ISGF3 complex members (STAT1, STAT2, IRF9), in TYK2 knockouts and in the kinase-dead TYK2^K923E^ mutant (Fig. 1c and Extended Data Fig. 2b). STAT3 and STAT5 knockouts led to downregulation of a subset of ISGs mainly in macrophages, indicating cooperative regulation of ISGs by STAT3 and STAT5 with ISGF3 members under homeostatic conditions (Extended Data Fig. 2b).

Transcriptional changes were often shared across two or more JAK-STAT mutants, indicative of synergy and cooperativity. However, we did not detect a single JAK-STAT signature that was consistently abrogated by all JAK-STAT knockouts (Extended Data Fig. 2c). Rather, each JAK-STAT protein appears to control a characteristic and cell-type-specific set of target genes (Supplementary Table 2). To visualize these effects across mutants and cell types, we performed dimensionality reduction with multi-dimensional scaling on the differentially expressed genes (Fig. 1d and Extended Data Fig. 2d,e). We observed a global separation in the transcriptional response for JAK-STAT knockouts with a primary role in the IFN response (STAT1, STAT2, IRF9, TYK2; green areas in Fig. 1d) versus those that are more strongly involved in cell maturation and differentiation (STAT3, STAT4, STAT5, STAT6; brown areas in Fig. 1d). Moreover, we identified six clusters with distinct properties (Fig. 1d, Extended Data Fig. 2d,e, Supplementary Table 2 and Supplementary Note).

In summary, our transcriptome analysis of 12 JAK-STAT mutant mouse models identified widespread and cell-type-specific gene-regulatory roles of JAK-STAT pathway members in homeostatic immune cells.

### Shared and specific gene modules regulated by JAK-STAT

**Fig. 2:**
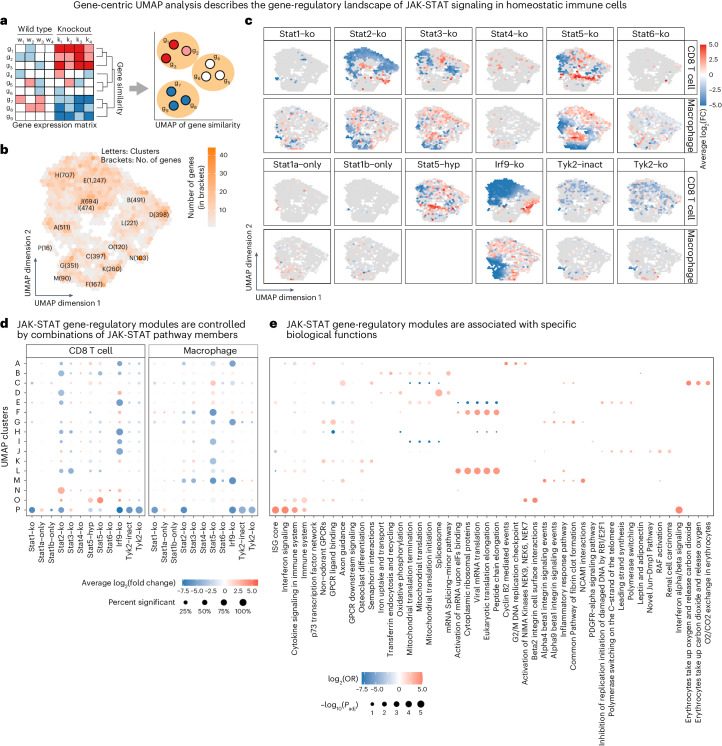
Gene-regulatory modules underlying JAK-STAT signaling in homeostasis. **a**, Outline of the analytical approach for identifying JAK-STAT gene-regulatory modules. **b**, Similarity of genes in terms of their differential expression patterns across JAK-STAT mutants, based on a UMAP of log_2_FCs between JAK-STAT mutant and matched wild-type samples. This UMAP places genes with similar effects of JAK-STAT mutants on their transcriptome in proximity. It includes all genes with a twofold or greater change in gene expression for at least one mutant, and it places them in 16 gene clusters marked by letters A to P. **c**, Overlay of mutant-specific differential expression (with color-coded log_2_FCs) on the gene UMAP from **b**. **d**, Dot plot showing the average log_2_FC across all genes in the clusters from **b**, for two cell types and 12 JAK-STAT mice. **e**, Dot plot showing gene set enrichment for the gene clusters from **b** (two-sided Fisher’s exact test, corrected for multiple comparisons). The four most enriched gene sets are shown for each cluster. OR, odds ratio.

For a comprehensive picture of JAK-STAT-mediated transcription regulation in homeostasis, we grouped all differentially expressed genes (*n* = 6,247) into gene-regulatory modules across mutants and cell types (Fig. 2a), establishing a transcriptional similarity map of genes using the Uniform Manifold Approximation and Projection (UMAP) algorithm. This method is widely used to visualize the similarity of single cells or samples, but here we applied it to visualize the similarity of effects on genes across mutants and cell types, to define regulatory modules. Based on the nearest neighbor graph from the UMAP algorithm, we clustered differentially expressed genes into 16 gene-regulatory modules that are regulated by JAK-STAT proteins (Fig. 2b and Supplementary Table 2). For each of these gene modules, we determined the average change in gene expression in each JAK-STAT mutant (Fig. 2c,d), and we annotated each module with its putative biological functions based on characteristic gene set enrichments (Fig. 2e).

**Extended Data Fig. 3:**
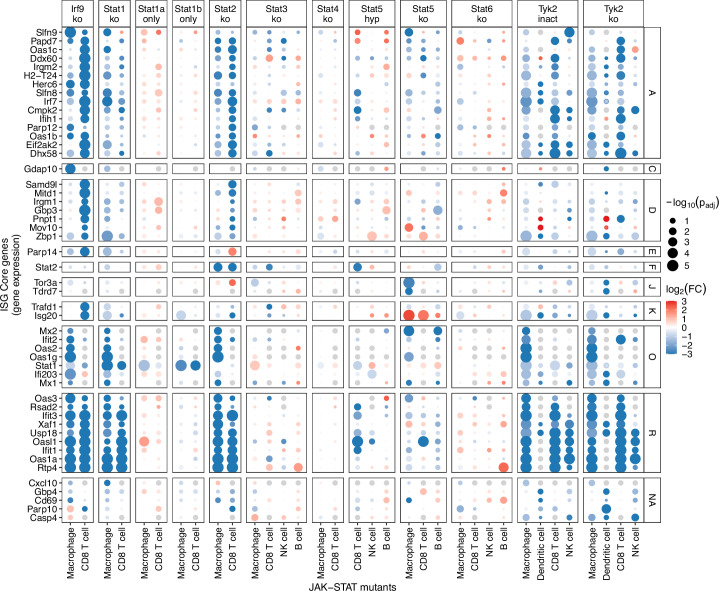
Differential expression of core ISGs between JAK-STAT mutant and wildtype mice. Dot plot showing log_2_FCs for the 50 genes with the largest log_2_FC (two-sided linear mixed models, corrected for multiple comparisons). Genes are grouped based on the clusters derived from the UMAP analysis. padj: adjusted p-value.

Our analysis identified a gene cluster (module P) that was highly enriched for the previously described ‘ISG core’ gene set^28^ (Extended Data Fig. 3). This module was strongly downregulated in knockouts of all three ISGF3 members (STAT1, STAT2, IRF9), in TYK2 knockouts and in the kinase-dead TYK2^K923E^ mutant, implicating these factors in tonic IFN signaling and baseline ISG expression in homeostatic immune cells^39,40^. STAT2-dependent gene expression was associated with ‘Oxidative phosphorylation’ and ‘mRNA-splicing’ (module D) in T cells but not in macrophages. IRF9 knockout increased expression of ‘Activation of NIMA kinases’ and ‘Beta2 integrin cell surface interactions’ in T cells (module O) and macrophage-specific downregulation of module M, which was associated with ‘beta1 integrin signaling’ and ‘NCAM1 interactions’.

Downregulation of module M was also observed in STAT5 knockout mice, indicative of cooperativity between IRF9 and STAT5. Moreover, IRF9 knockouts were characterized by increased expression of module F, which was enriched for ribosomal function and regulation of translation. STAT2 and IRF9 knockouts shared a pronounced effect on core ISGs that was consistent across cell types and appears to constitute a context-independent regulatory mechanism. However, core ISGs accounted only for a minority of their target genes, and other affected genes (including genes involved in cell differentiation and in broad IFN response signatures) showed much more cell-type-specific patterns (Supplementary Fig. 5). Overall, these observations suggest diverse regulatory effects of IRF9 that are independent of its established role as a member of the ISGF3 complex^20,41,42^.

STAT5 knockout affected several modules in cell-type-specific ways. Most notably, module B was downregulated in T cells but not in macrophages, while downregulation of module F was much more pronounced in macrophages than in T cells (Fig. 2d). We also observed upregulation of module O in T cells, which was associated with cell cycle regulators such as NIMA-related kinases (NEK1, NEK2) (Fig. 2e). Hyperactivated STAT5B^N642H^ had a less pronounced effect on modules G, J and N than STAT5 knockout, while affecting a broader range of other modules. It thus seems that this oncogenic variant of STAT5 has lost much of the conventional regulatory effects of the wild-type protein while having acquired many new target genes.

Our module-based analysis thus revealed diverse and often cell-type-specific regulatory processes and target genes in homeostatic immune cells, of which classical ISGs constitute only a small fraction. In addition, these results uncovered a much broader and more independent role for IRF9 than previously appreciated.

### Effect of JAK-STAT isoforms and mutations on immune cells

**Fig. 3:**
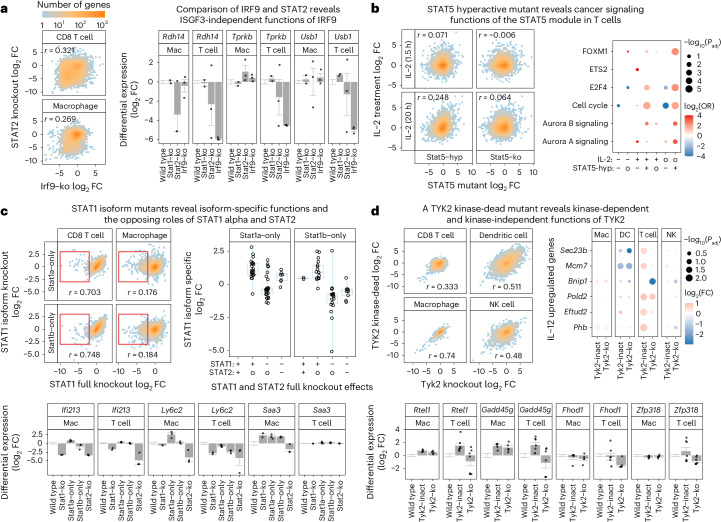
Characteristic roles of JAK-STAT signaling in homeostasis. **a**, Differential gene expression for IRF9 and STAT2 knockouts. Left, scatterplot of log_2_FCs for the two knockouts relative to matched wild-type samples. Right, bar plots of differential expression levels relative to wild type for selected genes, displaying mean and standard error. **b**, Differential gene expression for STAT5 modulation and IL-2 treatment in T cells. Left, scatterplot of log_2_FCs for the hyperactivated STAT5B^N642H^ mutant (STAT5-hyp) and STAT5 knockout relative to wild type, and for the response of wild-type T cell to in vitro IL-2 treatment at two time points. Right, gene set enrichment analysis for the differentially expressed genes (two-sided Fisher’s exact test, corrected for multiple comparisons). Upregulation, downregulation and no change are indicated by ‘+’, ‘−’ and ‘o’, respectively. **c**, Differential gene expression for STAT1 isoforms. Left, scatterplot of log_2_FCs for the two STAT1 isoforms and for the full STAT1 knockout. Right, box plots showing STAT1 isoform effects (log_2_FC) for genes with significant STAT1 effect in macrophages, grouped by the effects of full STAT1 and STAT2 knockouts. Upregulation, downregulation and no change are indicated by a ‘+’, ‘−’ and ‘o’, respectively. Box plots show the full data range, with the box indicating interquartile range and median. Bottom, bar plots showing the expression levels of selected genes affected by these mutants. **d**, Differential gene expression for TYK2 modulation. Left, scatterplot of log_2_FCs for the TYK2 knockout and the kinase-dead TYK2^K923E^ mutant. Right, TYK2 mutant effects on selected IL-12 regulated genes (two-sided linear mixed models, corrected for multiple comparisons). Bottom, bar plots showing the expression levels of selected genes affected by these mutants. Mac, macrophage; *r*, Spearman correlation coefficient. Bar plots display the mean and standard error.

**Extended Data Fig. 4:**
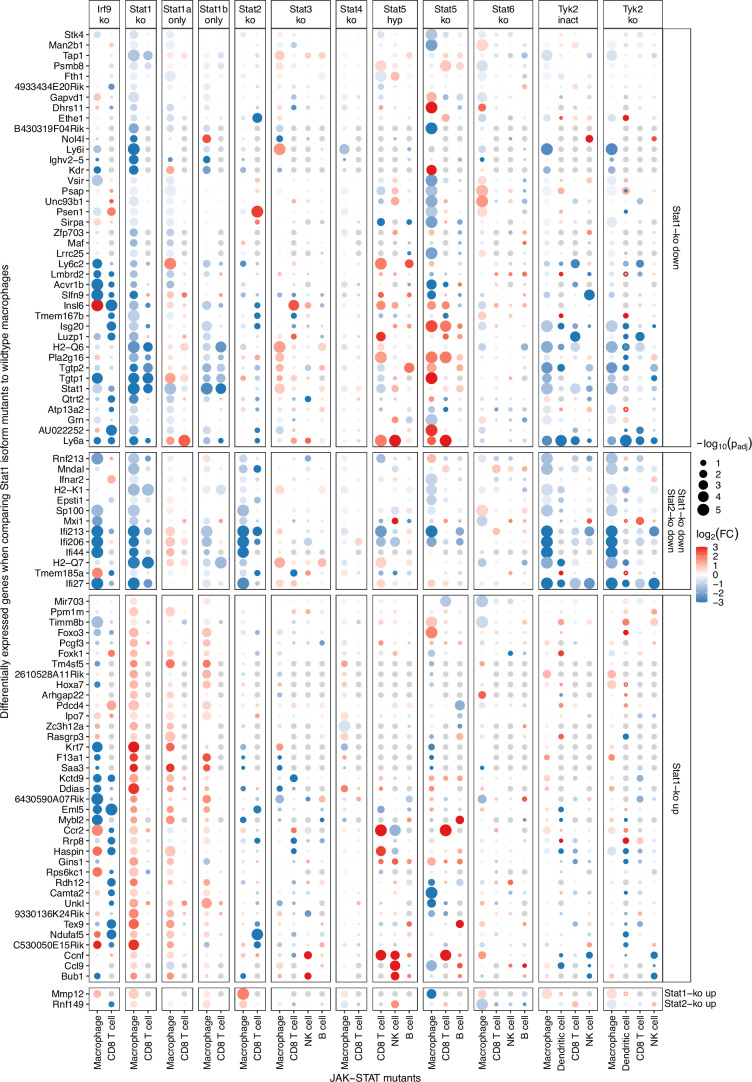
Differential expression of genes regulated by STAT1 isoform-only mutants in macrophages. Dot plot showing log_2_FCs for STAT1 isoform-only regulated genes grouped based on STAT1 and STAT2 mutant effects in macrophages (two-sided linear mixed models, corrected for multiple comparisons). padj: adjusted p-value.

The homeostasis-linked gene modules (Fig. 2) comprise many target genes of JAK-STAT signaling with well-known roles in active immune responses. However, we also observed characteristic differences and properties that appear to be specific to homeostatic immune cells. Here we focus on four examples (two are summarized below and two in the Supplementary Note): broad effects of STAT2 and IRF9 knockouts (Fig. 3a), dramatic changes in the specificity of hyperactivated STAT5B^N642H^ (Fig. 3b), differences between the two STAT1 splicing isoforms (Fig. 3c and Extended Data Fig. 4) and kinase-independent effects of TYK2 (Fig. 3d).

IRF9 and STAT2 are known for their role in the IFN response as part of the ISGF3 complex, but we observed much broader and only weakly correlated transcriptional changes for STAT2 and IRF9 knockouts in homeostatic immune cells (Figs. 2c and 3a). For example, IRF9 appears to regulate the following genes independent of STAT1 and the ISGF3 complex: *Rdh14*, important for signaling downstream of the retinoic acid receptor^43^; *Tprkb*, a critical component for the generation of transfer RNAs with a known role in p53-deficient cancers^44^; and *Usb1*, which is involved in hematopoietic malignancies^45^ (Fig. 3a). Our analyses demonstrate that IRF9 regulates many of its target genes independent of STAT1, STAT2 and of the canonical ISGF3 complex, possibly by interacting with other transcription factors including members of the STAT family^46,47^. The transcriptional changes observed in IRF9 knockouts showed a high correlation with those found in STAT3 and STAT5 knockout macrophages (Extended Data Fig. 2e), suggesting STAT3 and STAT5 as potential interaction partners of IRF9 in macrophages under homeostatic conditions.

We also investigated STAT5 knockout and STAT5B^N642H^ (Stat5-hyp) mutants with additional experiments. In canonical JAK-STAT signaling, STAT5 is activated in response to IL-2 signaling in T cells, prompting us to treat splenic T cells from wild-type, STAT5 knockout and Stat5-hyp mutant mice with IL-2. We assessed the effects of IL-2 treatment after 1.5 and 20 h and observed pronounced differences between STAT5 wild-type and knockout T cells (Fig. 3b), with correlations close to zero (Spearman’s *r* = −0.006 at 1.5 h; Spearman’s *r* = 0.064 at 20 h). STAT5 knockout thus compromises the gene-regulatory program associated with IL-2 stimulation. Surprisingly, STAT5B^N642H^-mutant T cells also showed little overlap with STAT5 wild type upon IL-2 stimulation (Spearman’s *r* = 0.071 at 1.5 h; Spearman’s *r* = 0.248 at 20 h), suggesting that this oncogenic driver mutation compromises normal STAT5 function and redirects the regulatory activity. Gene set analysis identified enrichment for AURORA kinase signaling (in line with a recent observation^48^), cell cycle progression and target genes of the transcription factors E2F4 and FOXM1 (Fig. 3b). These results suggest a switch of target genes for the STAT5B^N642H^ mutant compared with STAT5 wild type, which likely contributes to its role in T cell proliferation and lymphoma/leukemia development.

These results illustrate the breadth and complexity of JAK-STAT-mediated gene regulation under homeostatic conditions, which diverges in part from our knowledge of JAK-STAT signaling in active immune responses. Most notably, we found widespread IRF9-regulated gene expression independent of STAT1 and STAT2, and a switch to de novo gene targets for the STAT5B^N642H^ driver oncogene.

### Baseline JAK-STAT signaling in the in vivo tissue context

A key result of our study is the unexpected breadth and complexity of baseline JAK-STAT signaling under homeostatic conditions, which we observed in T cells and macrophages extracted from the spleens of unperturbed mice. To exclude that this effect is due to sample handling rather than reflecting true biology (for example, tissue dissociation may activate immune cells), we investigated the expression of JAK-STAT target genes directly in spleen tissue using spatial transcriptomics (Visium assay) and RNA-based fluorescence in situ hybridization (RNA-FISH), without any cell isolation or fluorescence-activated cell sorting (FACS).

**Fig. 4:**
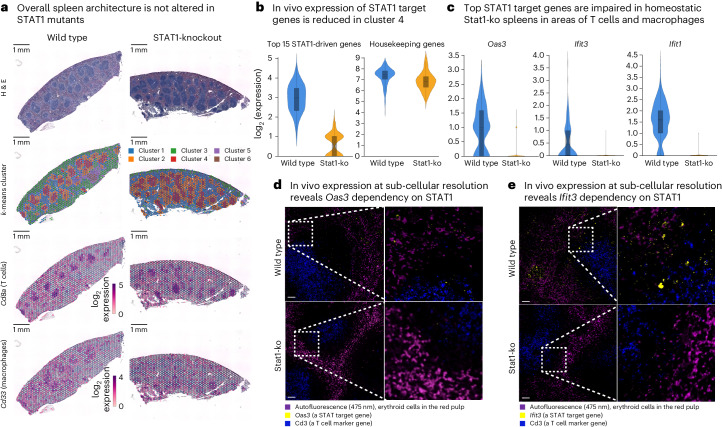
In vivo validation of baseline JAK-STAT signaling in homeostasis. **a**, Spatial transcriptomics profiles of spleens from wild-type and STAT1 knockout mice, shown for samples collected after in vivo cell fixation using formaldehyde. First row: hematoxylin and eosin (H&E) stains highlighting the anatomical structures of the spleen. Second row: spatial transcriptomics profiles annotated with gene expression clusters. Third and fourth row: expression levels of the T cell marker gene *Cd8a* and the macrophage marker gene *Cd33* in the spatial transcriptomics data (scale bars, 1 mm). **b**, Violin plots showing the expression of STAT1-driven genes (top-15 downregulated genes comparing STAT1 knockout and wild type based on the RNA-seq data) and housekeeping genes (*Actb*, *Hprt* and *Ubc*) in the spatial transcriptomics data. **c**, Violin plots showing the expression of the ISGs *Oas3*, *Ifit3* and *Ifit1* in Cluster 4 of the spatial transcriptomics data. **d**, Representative RNA-FISH images for the ISG *Oas3* (yellow) and the T cell marker gene *Cd3e* (dark blue) in spleen samples from wild-type and STAT1 knockout mice (scale bar, 50 µm). Autofluorescence of the red pulp is visible in magenta. **e**, Representative RNA-FISH images for the ISG *Ifit3* (yellow) and the T cell marker *Cd3e* (dark blue) in spleen samples from wild-type and STAT1 knockout mice (scale bar, 50 µm). Experiments comprised two mice (**a**–**c**) or three mice (**d** and **e**) as biological replicates. Box plots (**b** and **c**) show the full data range, with the box indicating the interquartile range and median.

**Extended Data Fig. 5:**
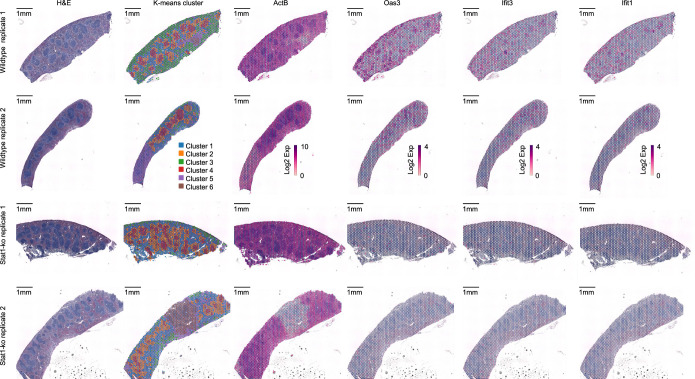
Spatial transcriptomics profiles of spleens from wildtype and STAT1 knockout mice. Visualization of transcriptomics profiles for spleen samples from wildtype and STAT1 knockout mice (two biological replicates) that were formaldehyde-fixed in vivo, sliced and stained with H&E (first column; partially overlapping with plots presented in Fig. 4). K-means clustering identified six gene expression clusters (second column; partially overlapping with plots presented in Fig. 4). Spatial distribution of gene expression is further shown for the housekeeping gene *Actb* (third column) and the ISGs *Oas3*, *Ifit3*, and *Ifit1* (fourth to sixth column).

For spatial transcriptomics profiling, we fixed spleens from wild-type and STAT1 knockout mice in situ via transcardial perfusion with formaldehyde, which effectively removes the risk of altering gene expression during ex vivo sample handling. The spatial transcriptomics data reflected the expected architecture of the spleen in both wild-type and knockout mice (Fig. 4a and Extended Data Fig. 5). The *k*-means clustering of the spatially resolved transcriptional profiles identified six clusters, four of which (Clusters 1 to 4) corresponded to well-known morphological regions of the spleen, including areas of white and red pulp. The overall tissue architecture was unaffected by the STAT1 knockout, and the localization of T cells (marked by *Cd8a* expression) and macrophages (marked by *Cd33* expression) was similar between wild-type and knockout mice.

We then quantified the expression of the top-15 downregulated genes between STAT1 knockout and wild-type mice (based on our RNA-seq data) in the spatial transcriptomics data. We observed significantly higher expression of these putative STAT1 target genes in wild-type compared with STAT1 knockout mice specifically for spatial Cluster 4, which corresponds to white pulp—an area that contains many T cells and macrophages (Fig. 4b and Extended Data Fig. 5). For example, the classical ISGs *Oas3*, *Ifit3* and *Ifit1* were expressed in wild-type mice but almost completely absent in STAT1 knockouts (Fig. 4c). In contrast, a control gene signature comprising the putative housekeeping genes *Actb*, *Hprt* and *Upc* showed similar expression levels between wild type and STAT1 knockouts (Fig. 4b and Extended Data Fig. 5).

Given that the resolution of the spatial transcriptomics assay does not support single-cell analysis, we further validated these results using single-molecule RNA-FISH for *Oas3* and *Ifit3* (Fig. 4d,e). Consistent with the spatial transcriptomics data, we observed *Oas3* and *Ifit3* expression in wild-type mice but not in STAT1 knockout mice, both for the spleen’s white pulp (which is marked by Cd3-expressing T cells) and the red pulp (marked by erythrocyte-mediated autofluorescence).

These results show that baseline JAK-STAT signaling under homeostatic conditions is an in vivo characteristic of splenic immune cells in wild-type mice and is abrogated in STAT1 knockout mice.

### JAK-STAT chromatin regulation in homeostatic immune cells

**Fig. 5:**
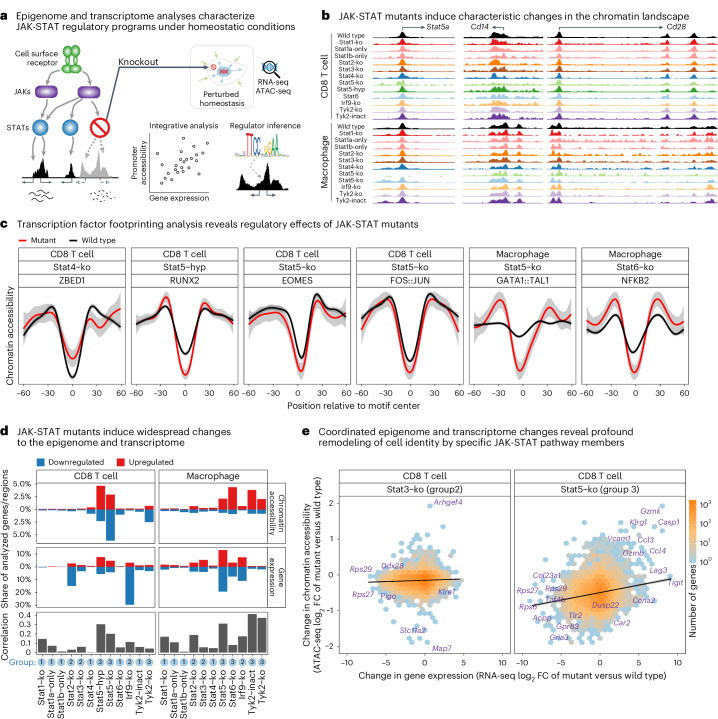
Epigenome effects of JAK-STAT mutants in homeostasis. **a**, Outline of the analysis dissecting JAK-STAT modulation of the epigenome (based on ATAC-seq data) and transcriptome (based on RNA-seq data). **b**, Genome browser tracks showing chromatin accessibility profiles for the promoter regions of the *Stat5a* gene, the macrophage marker gene *Cd14* and the T cell marker gene *Cd28*. **c**, Transcription factor footprinting analysis, showing differential chromatin accessibility footprints for certain JAK-STAT mutants and cell types. Shaded areas indicate the standard error. **d**, Bar plots showing the percentage of genes and genomic regions affected by transcriptome and epigenome changes upon JAK-STAT modulation (relative to the number of all tested genes or genomic regions), as well as the Pearson correlation between transcriptome and epigenome changes (log_2_FCs of gene expression versus chromatin accessibility of the corresponding gene promoter across all tested genes). Group annotations (in blue) were manually assigned based on qualitative similarities in the transcriptome and epigenome effects. **e**, Scatterplot of log_2_FCs for transcriptome versus epigenome changes upon STAT3 and STAT5 knockout.

**Extended Data Fig. 6:**
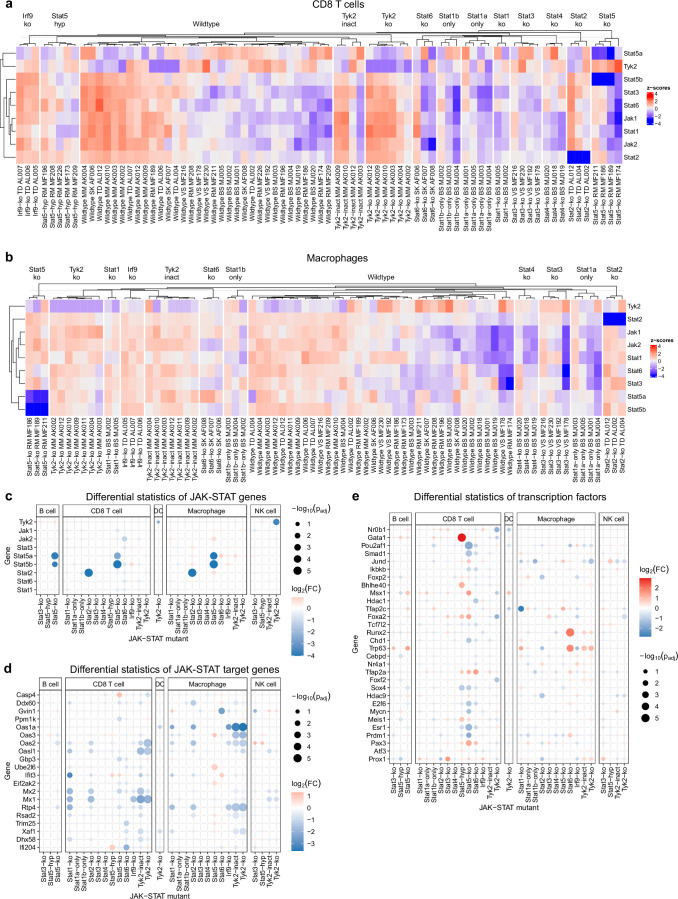
JAK-STAT modulated chromatin accessibility at promoters of transcriptional regulator genes. (**a**, **b**) Chromatin accessibility (ATAC-seq signal) transformed to z-scores for JAK-STAT genes in CD8^+^ T cells (**a**) and macrophages (**b**). (**c**–**e**) Differential accessibility results for JAK-STAT genes (**c**), JAK-STAT target genes (**d**), and other transcriptional regulators (**e**). padj: adjusted p-value. P-values in panels c-e are based on two-sided linear mixed models corrected for multiple comparisons.

Epigenetic mechanisms play an important role in the regulation of cell state^49,50^, and JAK-STAT is known to induce changes to the epigenome upon acute immune stimulation^28,29,51–54^. We thus hypothesized that baseline JAK-STAT activity helps maintain the ‘epigenetic potential’ of immune cells^55–58^, by keeping immune cells in a regulatory state that supports rapid activation without previous chromatin remodeling. We investigated the effect of perturbed JAK-STAT signaling on chromatin accessibility (ATAC-seq) for the same JAK-STAT mutants and cell types as in the transcriptome analysis (Fig. 5a, Extended Data Fig. 6 and Supplementary Table 1). These epigenome maps showed mutant-specific as well as cell-type-specific differences (Fig. 5b). For example, chromatin accessibility of the *Stat5a* gene promoter was reduced in STAT5 knockout macrophages and T cells, indicative of abrogated feed-forward regulation, and an intronic region of *Cd28* carried accessible chromatin only in T cells, whereas an upstream enhancer of the *Cd14* gene was accessible only in macrophages.

To link the affected genomic regions to putative regulators, we inferred transcription factor binding from DNA sequence motifs (Fig. 5c). We identified enriched binding sites of RUNX2 in STAT5B^N642H^ mutant T cells, EOMES and AP1 heterodimer (FOS/JUN) in STAT5 knockout T cells, GATA1/TAL1 in STAT5 knockout macrophages and NFκB in STAT6 knockout macrophages—all associated with regions with increased chromatin accessibility in the JAK-STAT mutants. Conversely, binding sites of ZBED1, which regulates cell proliferation^59^, were enriched in regions with decreased chromatin accessibility in STAT4 knockout T cells.

**Extended Data Fig. 7:**
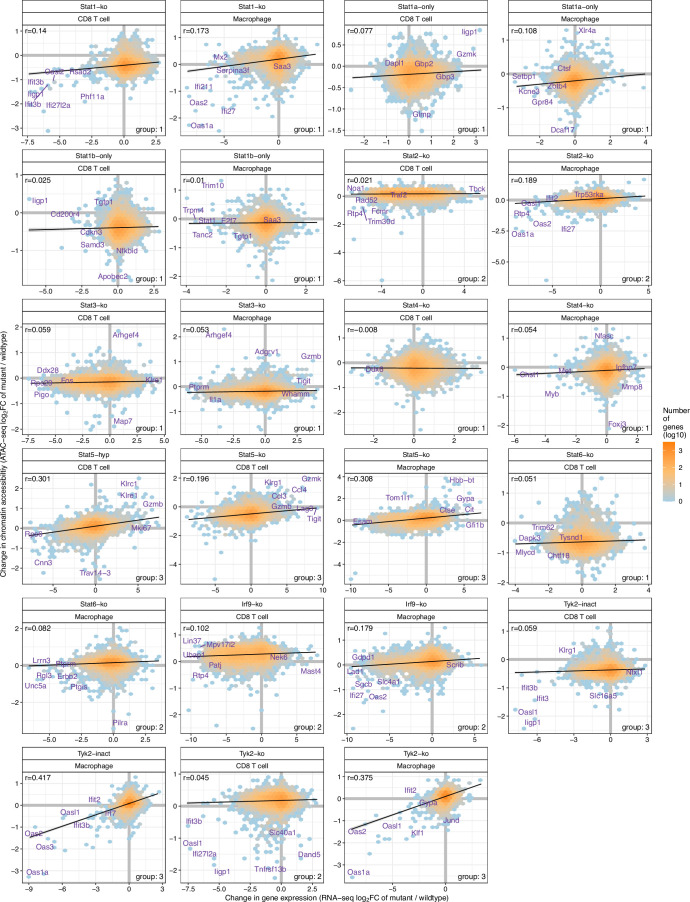
Comparison of transcriptome versus epigenome changes for each of the JAK-STAT mutants. Scatterplots of log_2_FCs for differential gene expression and differential promoter chromatin accessibility across the twelve JAK-STAT mutants. The Pearson correlation (r) is indicated in each plot.

**Extended Data Fig. 8:**
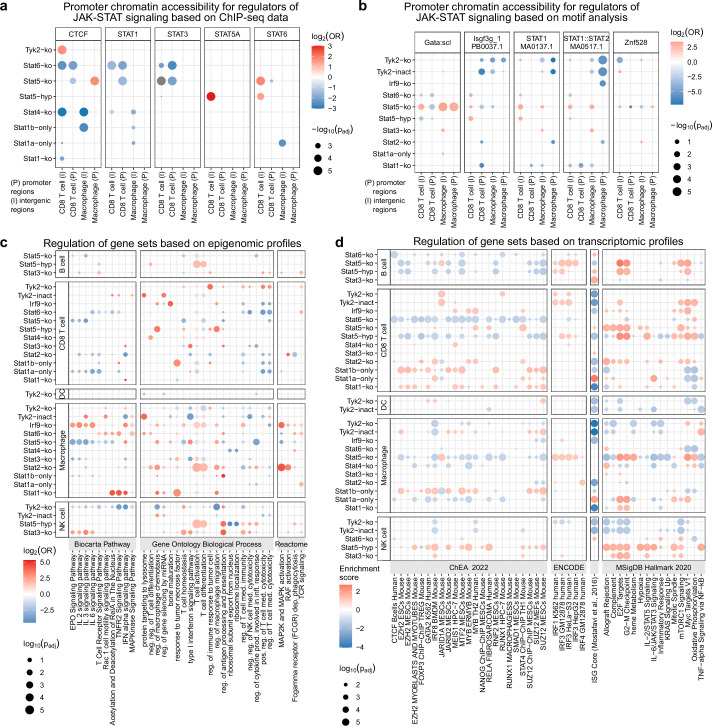
Integrative analysis of JAK-STAT mutant effects on the transcriptome and epigenome. (**a**) Enrichment of regulator binding regions (from public ChIP-seq datasets) in differential chromatin regions (two-sided Fisher’s exact test, corrected for multiple comparisons). Grey dots represent examples where both enrichment and depletion were identified for different ChIP-seq datasets. (**b**) Enrichment of regulator binding profiles (from transcription factor binding motif analyses) in differential chromatin regions (two-sided hypergeometric test, corrected for multiple comparisons). (**c**) Enrichment of gene sets in differential chromatin regions (two-sided logistic regression, corrected for multiple comparisons). (**d**) Enrichment of gene sets in differentially expressed genes (two-sided chi squared test, corrected for multiple comparisons). OR: odds ratio; padj: adjusted p-value.

Moreover, to quantify the effects of JAK-STAT proteins on the epigenomes of homeostatic immune cells, we systematically compared the chromatin accessibility profiles between JAK-STAT mutant and wild-type mice (Supplementary Table 3). Mutants with many differences in their epigenomes also tended to differ strongly in their transcriptomes, although the association was far from perfect (Fig. 5d). We further compared the JAK-STAT mutant effects on promoter accessibility with those on gene expression across genes (Fig. 5d,e and Extended Data Figs. 7 and 8). Correlations ranged from zero to above 0.4, and we identified three groups of JAK-STAT mutants with distinct patterns (Fig. 5d and Supplementary Note).

The first group comprised knockouts of STAT1 and its two isoforms (in both cell types), STAT4 (in both cell types) and STAT6 (in T cells) and the kinase-dead TYK2^K923^ mutant (in T cells). These mutants induced relatively few changes to the epigenome (<2.5% of tested regions) and to the transcriptome (<4.5% of genes), and the changes were not well correlated (Pearson’s *r* < 0.2).

The second group of JAK-STAT mutants was characterized by many transcriptional changes (>4.5% of genes) but fewer epigenome changes (<2.5% of tested regions), and limited correlation between the two (Pearson’s *r* < 0.2). This group included knockouts of IRF9, STAT2 and STAT3 (in both cell types). STAT2 and IRF9 knockouts led to decreased expression of ISGs such as *Oasl1*, *Ifit2*, *Ifi27*, *Oas1a*, *Oas2* and *Lad1*. Moreover, STAT3 knockouts caused widespread transcriptional changes but only modest changes of the epigenome, despite STAT3’s essential role as a developmental regulator.

The third group was characterized by a stronger effect on the epigenome (>2.5% of tested regions). This group included the hyperactivating STAT5B^N642H^ mutant (in T cells), knockouts of STAT5 (in both cell types), STAT6 (in macrophages) and TYK2 (in both cell types) and the kinase-dead TYK2^K923^ mutant (in macrophages). These mutants (except for TYK2) also exhibited strong transcriptome effects (>4.5% of tested genes) and a relatively high correlation of epigenome and transcriptome. Knockouts of STAT5 and STAT6 resulted in increased chromatin accessibility specifically in macrophages, indicative of a repressive role of these factors under homeostatic conditions and in line with known STAT6-mediated repression of M1 polarization genes^25^ (Fig. 5e). The oncogenic STAT5B^N642H^ mutant lost the repressive effect of STAT5 and instead caused T cell-specific increased chromatin accessibility, as well as upregulation of T cell effector genes (granzymes *Gzmk, Gzmb*), of killer cell lectin-like receptors (*Klrc1*, *Klre1*) and of the cell cycle regulator *Mki67*, which likely contributes to hyperproliferation of STAT5B^N642H^ T cells.

Integrative epigenome and transcriptome analysis thus identified chromatin-regulatory roles of multiple JAK-STAT pathway members, which were not always linked to changes in gene expression. Our observations suggest that baseline JAK-STAT signaling under homeostatic conditions actively maintains a chromatin accessibility landscape that supports rapid immune responses—but carries the risk of oncogenic transformation, as illustrated by the changes associated with STAT5B^N642H^ and the well-established oncogenic role of this mutant.

### Loss of JAK-STAT signaling upon removal of tissue context

**Fig. 6:**
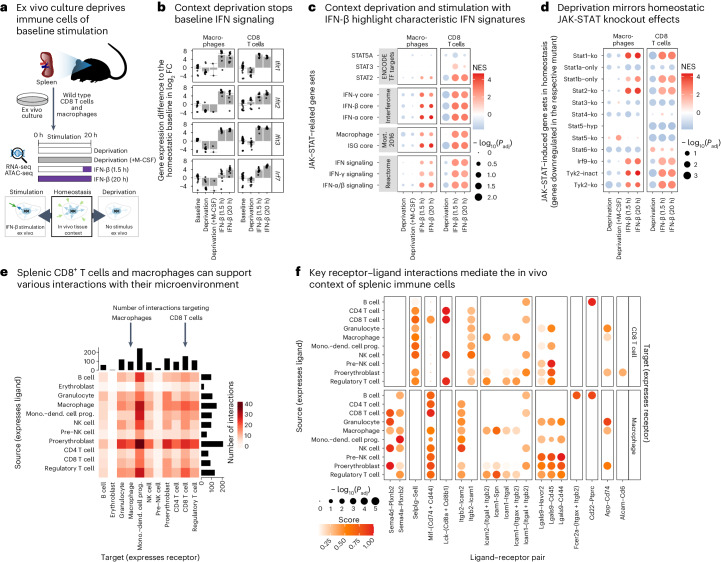
Abrogated baseline JAK-STAT signaling outside of the in vivo tissue context. **a**, Outline of the experimental approach: ex vivo culture for 20 h with basal medium and 10% FCS without supplements or with M-CSF (to support macrophage viability) or with IFN-β stimulation either in the last 1.5 h before sample collection or for the full 20 h, followed by transcriptome profiling. **b**, Differential expression upon ex vivo culture compared with homeostatic conditions in wild-type cells. Bar plots display the mean and standard error of log_2_FCs. **c**, Enrichment or depletion of JAK-STAT-related gene sets among the differentially expressed genes from **b**. **d**, Enrichment or depletion of differentially expressed genes between JAK-STAT mutants and wild type from in vivo homeostatic conditions among the differentially expressed genes from **b**. For example, enrichment (red) of STAT1 knockout genes for IFN-β stimulation indicates that our homeostatic STAT1 target genes are preferentially induced by IFN-β stimulation. **e**, Summary of inferred receptor–ligand interactions in the spleen as inferred from the Tabula Muris dataset. Interactions where CD8^+^ T cells and macrophages represent targets (that is, express the receptor) are highlighted by black arrows. **f**, Selected ligand–receptor interactions of CD8^+^ T cells (top) and macrophages (bottom) with other types of immune cells. NES, normalized enrichment scores. *P* values in **c**, **d** and **f** are based on two-sided random sampling, corrected for multiple comparisons.

Our analyses uncovered widespread changes in the transcriptomes and epigenomes of homeostatic immune cells obtained from the spleen of JAK-STAT mutant mice, strongly suggestive of baseline JAK-STAT signaling in wild-type mice in the absence of acute immune stimuli. We hypothesized that this baseline JAK-STAT signaling under homeostatic conditions is triggered by the in vivo tissue context of the immune cells. To test this hypothesis, we deprived T cells and macrophages of their tissue context through short-term ex vivo culture, effectively removing them from interactions with other cell types and from secreted factors that may trigger baseline JAK-STAT signaling activity in intact tissue (Fig. 6a). In addition, we stimulated the ex vivo-cultured cells with IFN-β to actively induce JAK-STAT activity, and we conducted a control experiment in which we supplied macrophages only with the macrophage colony stimulating factor M-CSF to enhance their tolerance for ex vivo culture. We profiled the transcriptomes and epigenomes of all samples and compared the results with wild-type cells purified from homeostatic tissue samples (Supplementary Tables 4 and 5).

**Extended Data Fig. 9:**
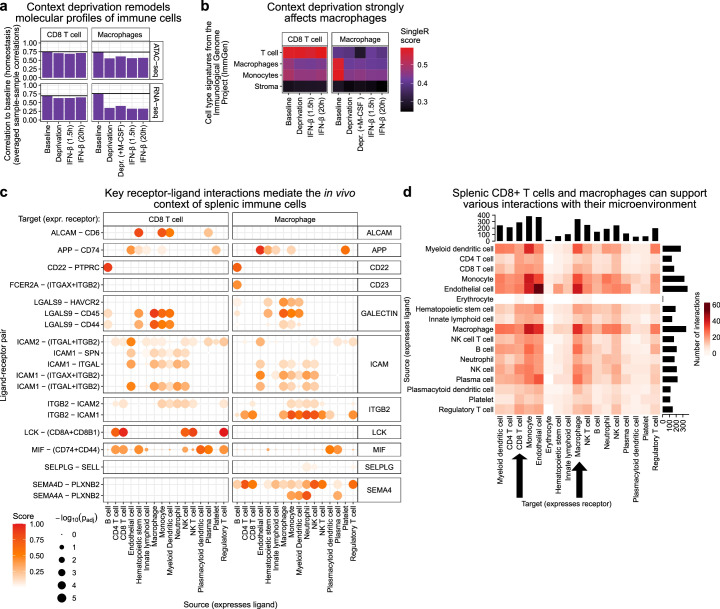
Loss of baseline JAK-STAT signaling outside of the in vivo tissue context. (**a**) Average sample-to-sample Spearman correlation among homeostatic, context-deprived and stimulated immune cells, visualizing the effects of ex vivo culture on wildtype immune cells. The average correlation among samples under homeostasis is shown as baseline indicated by a black line. (**b**) SingleR similarity scores^110^ comparing cultured and homeostatic cells to external reference profiles from the ImmGen consortium^111^. (**c**, **d**) Inferred receptor-ligand interactions of T cells and macrophages in single-cell transcriptome data from Tabula Sapiens. Examples of ligand-receptor interactions (**c**) between CD8^+^ T cells (left), macrophages (right) and various splenic cells (rows) (two-sided random sampling, corrected for multiple comparisons), and the total number of receptor-ligand interactions inferred from Tabula Sapiens (**d**). padj: adjusted p-value.

Deprivation of tissue context by ex vivo culture resulted in strong downregulation of genes (Fig. 6b) and pathways (Fig. 6c) related to JAK-STAT and IFN signaling, both in T cells and in macrophages. In contrast, IFN-β stimulation upregulated these gene signatures well above homeostatic levels (Fig. 6b,c). These effects were robust across biological replicates and strongly exceeded technical variation in our dataset (Supplementary Figs. 6 and 7). The transcriptional changes observed in T cells were consistent with switching between different levels of JAK-STAT signaling activity based on extrinsic signaling input. In contrast, macrophages depleted of their tissue context not only exhibited widespread loss of JAK-STAT-mediated gene expression, but also a broader downregulation of macrophage-specific gene expression programs (Extended Data Fig. 9a,b). Neither IFN-β stimulation nor treatment with macrophage growth factor M-CSF was able to rescue this wider loss of macrophage-specific gene expression programs. In other words, both T cells and macrophages depended on signals from the in vivo tissue context to maintain baseline JAK-STAT signaling activity, but only macrophages depended on the tissue context to maintain their cellular identity.

To assess which JAK-STAT proteins may mediate the stimulatory effect of the in vivo tissue context, we compared the differentially expressed genes for JAK-STAT pathway mutants (relative to wild type) with the differentially expressed genes for the ex vivo-cultured wild-type cells (relative to uncultured wild-type cells) (Fig. 6d). We found that target genes of STAT1 (including each of the two isoforms), STAT2, IRF9 and TYK2 (including its catalytically inactive mutant) were downregulated upon deprivation of tissue context in wild-type cells. Ex vivo stimulation with IFN-β rescued most of these effects, with the exception of STAT1-beta-dependent genes. In macrophages, context deprivation also led to the downregulation of STAT3-, STAT4- and STAT6-dependent genes, which was not rescued by IFN-β stimulation. Finally, STAT5-dependent genes were downregulated in cultivated T cells and not restored by IFN-β stimulation (Fig. 6d).

Our observation that baseline JAK-STAT signaling is lost in context-deprived ex vivo culture, but partially restored by IFN-β stimulation, suggests cell-extrinsic triggers of baseline JAK-STAT signaling under homeostatic conditions. In contrast, it excludes cell-intrinsic effects that would persist in cell culture (for example, accumulating DNA damage in adult mice) as the primary cause of baseline JAK-STAT signaling. To identify cell-extrinsic factors that may induce baseline JAK-STAT signaling in vivo, we inferred receptor–ligand interactions of T cells and macrophages with other cell types of the spleen, based on published single-cell transcriptome atlas data (Fig. 6e,f, Extended Data Fig. 9c,d and Supplementary Table 6)^60^. For example, splenic CD8^+^ T cells highly expressed the KLRB1 receptor, supporting cell–cell interactions with immune cells that express CLEC2B or other c-type lectins^61^. Splenic macrophages were characterized by high expression of checkpoint molecule receptors such as SIGLEC1 (which can interact with SPN on T cells)^62^ and LILRB1 (which can interact with HLA-F/MHC-I on many cell types)^63^. Moreover, the HAVCR2/TIM3-LGALS9 receptor–ligand pair^64^ may mediate macrophage interactions with most types of myeloid immune cells in the spleen.

These functional experiments show that removing T cells and macrophages from their in vivo tissue context abrogates the baseline JAK-STAT activity that we found to be characteristic of homeostatic immune cells. Deprivation of tissue context mimicked the effect of certain JAK-STAT pathway knockouts and was partially rescued by the strong exogeneous stimulation provided by IFN-β, suggesting that low-level IFN signaling and ISGF3 activity are important contributors to homeostatic JAK-STAT signaling.

### IFN-β partially rescues JAK-STAT signaling in mutant cells

**Fig. 7:**
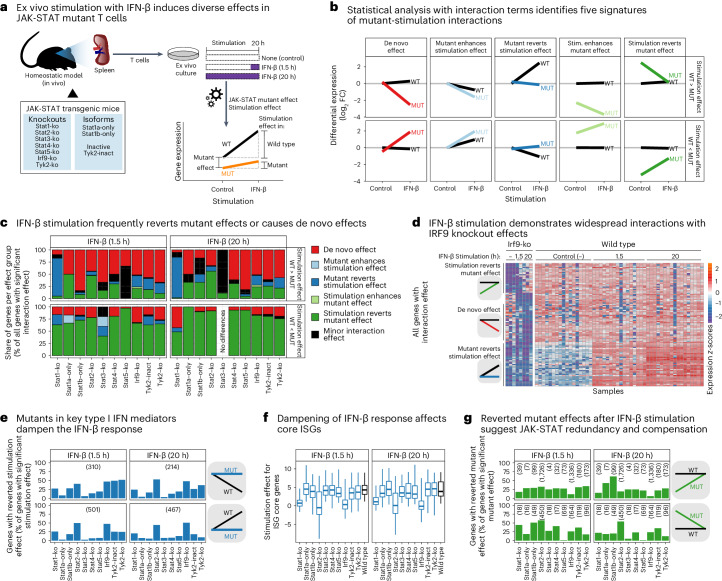
Partial restoration of wild-type signaling upon stimulation of JAK-STAT mutant T cells. **a**, Outline of IFN-β stimulation experiments and analyses in JAK-STAT knockout cells cultured ex vivo. This figure focuses on T cells, while corresponding results for macrophages are shown in Extended Data Fig. 10. **b**, Grouping of genes based on the observed IFN-β stimulation effects in wild-type and mutant cells. Lines correspond to the mean transcriptional change across all genes in each group. **c**, Prevalence of the five gene groups from **b** in each JAK-STAT mutant. Genes with significant but minor differences of stimulation effects between wild-type and mutant cells were not assigned to any group (marked in black). **d**, Differential gene expression heatmap for IRF9 knockout and wild-type T cells upon IFN-β stimulation, annotated with the grouping of differentially expressed genes (rows). **e**, Share of genes for which the JAK-STAT mutant effect reverts the IFN-β stimulation effect. This is calculated as the percentage of all genes with an IFN-β stimulation effect in wild-type cells, the total number of which is shown in brackets. **f**, Mean differential gene expression (log_2_FC) upon IFN-β stimulation across 80 core ISGs. Box plots show the full data range, with the box indicating interquartile range and median. **g**, Share of genes for which the IFN-β stimulation reverts the JAK-STAT mutant effect, relative to all genes with a JAK-STAT mutant effect in unstimulated cells (shown in brackets). MUT, mutant; WT, wild type.

**Extended Data Fig. 10:**
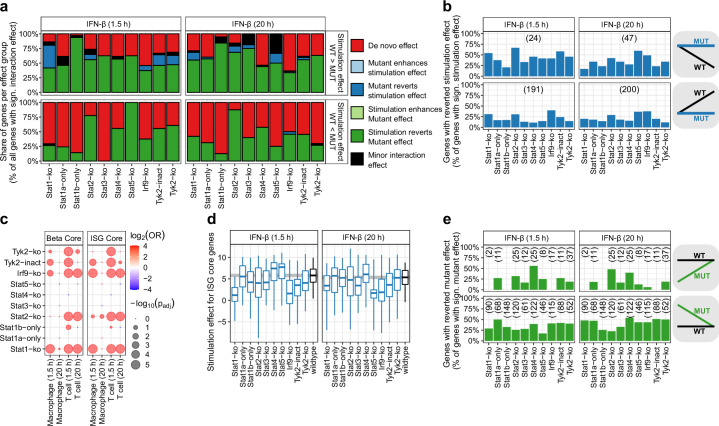
Partial restoration of baseline JAK-STAT signaling upon stimulation of JAK-STAT mutant macrophages. (**a**) Prevalence of the five gene groups from Fig. 7b in each JAK-STAT mutant. This figure focuses on macrophages, while similar results for T cells are shown in Fig. 7b. (**b**) Share of genes for which the JAK-STAT mutant effect reverts the IFN-β stimulation effect. This is calculated as the percentage of all genes with a IFN-β stimulation effect in wildtype cells, the total number of which is shown in brackets. (**c**) Enrichment of core ISGs and IFN-β target genes among genes for which JAK-STAT mutants revert stimulation effects (two-sided Fisher’s exact test, corrected for multiple comparisons). This panel is the only panel in this figure that shows results for both macrophages and T cells. (**d**) Mean differential gene expression (log_2_FC) upon IFN-β stimulation across 68 core ISGs. Box plots show the full data range, with the box indicating interquartile range and median. (**e**) Share of genes for which the IFN-β stimulation reverts the JAK-STAT mutant effect, relative to all genes with a JAK-STAT mutant effect in unstimulated cells (shown in brackets). OR: odds ratio; padj: adjusted p-value.

Given that deprivation of tissue context in wild-type cells mimicked certain JAK-STAT mutant effects (Fig. 6), we further tested whether IFN-β stimulation could restore JAK-STAT signaling activity not only in context-deprived wild-type but also in JAK-STAT mutant immune cells. We thus cultured JAK-STAT mutant cells in vitro and stimulated them with IFN-β (Fig. 7a), in the same way as for wild-type cells shown in Fig. 6. We focused this analysis primarily on T cells (Fig. 7) given their stronger response to IFN-β in wild-type cells (Fig. 6c), while observing similar yet weaker effects also for macrophages (Extended Data Fig. 10).

To compare the transcriptome response of IFN-β stimulation between wild-type cells and each JAK-STAT mutant, we fitted linear models with corresponding interaction terms (Fig. 7a). Based on these fitted models, we assigned the differentially expressed genes to five signatures (Fig. 7b and Supplementary Table 7): (1) de novo response to IFN-β stimulation in mutant cells that is not observed in wild-type cells; (2) enhanced effect of stimulation (difference of stimulated versus unstimulated) in mutant cells compared with wild type; (3) reduced effect of stimulation in mutant cells compared with wild type; (4) enhanced mutant effect (difference of mutant to wild type) in stimulated cells; and (5) reduced mutant effect in stimulated cells. In groups (2) and (3) the mutant effect is minor, while in groups (4) and (5) the stimulus effect is minor. Across comparisons, most genes fell into signatures (1), (3) and (5) (Fig. 7c).

De novo effects of IFN-β stimulation (signature 1, red line in Fig. 7b) comprise genes with transcriptional changes upon IFN-β stimulation specifically in JAK-STAT mutant cells. Signature 1 included the transcription factor *Eomes* downregulated in STAT2 knockout T cells; the SWI/SNF family member *Smarca4* downregulated in STAT2 and IRF9 knockout T cells (Fig. 7d); the *Gzma* gene, encoding the T cell effector molecule granzyme A, downregulated in TYK2 knockout T cells; and the transcription factor *Klf16* upregulated in TYK2 knockout T cells (Supplementary Table 7). Genes with a reverted stimulation effect in mutant cells (signature 3, dark blue line in Fig. 7b) were upregulated upon IFN-β stimulation in wild-type cells only, which included many target genes of the JAK-STAT pathway (Extended Data Fig. 10c). Signature 3 was most prominent for knockouts of STAT1, STAT2, IRF9 and TYK2 (Fig. 7e,f), and included ISGs such as *Mx1*, *Oas2* and *Cxcl10* (Supplementary Table 7). Finally, IFN-β stimulation partially reverted a negative effect of JAK-STAT pathway mutants (signature 5, dark green line in Fig. 7b), comprising many genes for almost all knockouts (Fig. 7g), for example, the chromatin remodeler *Chd6* and the lysine demethylase *Kdm3a* upregulated in IRF9 knockout T cells, as well as the *Kmt5b* gene encoding a lysine methyl transferase and the *Sfpq* gene encoding a HAT complex member and splicing factor upregulated in STAT2 knockout T cells (Supplementary Table 7).

In summary, IFN-β stimulation provided partial rescue of JAK-STAT-regulated gene expression in all tested JAK-STAT mutants, indicative of pathway redundancy and the ability of IFN signaling to restore expression of mutant-affected genes well beyond the core ISGs. The stimulation-induced restoration of wild-type gene expression was most pronounced for target genes of STAT2 and IRF9, which appear to have key roles in maintaining baseline JAK-STAT signaling in immune cells under homeostatic conditions.

## Discussion

JAK-STAT signaling is one of the most studied and best understood signaling pathways. It constitutes a prototypical example of how cells recognize external stimuli using cell surface receptors, process these inputs through kinase signaling and activate transcription factors that control specific sets of target genes. The rapid conversion of external signals into transcriptional responses makes JAK-STAT signaling an ideal mechanism for immune gene activation^14,65,66^ and response to certain developmental stimuli^67,68^. In contrast, its dependence on external stimuli makes JAK-STAT signaling a less obvious candidate for maintaining cellular homeostasis.

Here we described widespread JAK-STAT signaling activity in immune cells from the spleen of unperturbed mice, which was triggered by signals and cell–cell interactions provided by the in vivo tissue context. Baseline JAK-STAT signaling was essential for maintaining immune gene activity and chromatin accessibility, and in the case of macrophages, for maintaining cellular identity. These observations were based on transcriptome (RNA-seq) and epigenome (ATAC-seq) profiles for 12 JAK-STAT mutant mouse models in five immune cell types (mainly T cells and macrophages) and multiple conditions (sorted primary cells, ex vivo culture to deplete tissue context, and IFN-β stimulation in wild-type and mutant mice). This large dataset also establishes a broadly useful resource of the JAK-STAT pathway, which will provide motivation and guidance for further research into the homeostatic roles of JAK-STAT. Indeed, the scale and scope of this study might make it the most comprehensive epigenome/transcriptome dissection of one signaling pathway that has yet been performed, and a blueprint for profiling other signaling pathways in immunology and beyond. Using a comparative analytical approach across different mutant mice, cell types and stimuli, we uncovered both shared and specific patterns of JAK-STAT signaling between different STATs, between T cells and macrophages, and in response to IFN-β stimulation.

The picture emerges of an elaborate signaling pathway characterized by specialization and cooperativity. Homeostatic immune cells lacking different subunits of the ISGF3 complex (STAT1, STAT2 or IRF9) all showed downregulation of core ISGs, indicative of low-level canonical JAK-STAT signaling under homeostatic conditions. We also found aspects of baseline JAK-STAT signaling deviating from the pathway’s well-established response to immune stimulation. For example, STAT2 and IRF9 knockout resulted in transcriptome changes that were different from each other and independent of STAT1. These target genes were not typical ISGs but partially overlapped with genes regulated by STAT3 and STAT5. Moreover, our analysis identified STAT1 as a regulator of chromatin accessibility well beyond its role in the ISGF3 complex, particularly for macrophages.

We found that homeostatic JAK-STAT signaling collapsed when we deprived immune cells of their in vivo tissue context, and the resulting transcriptional changes showed similarities with those observed in certain JAK-STAT mutants, highlighting the important stimulatory role of the in vivo tissue context. We were able to restore baseline JAK-STAT signaling and partially rescued its transcriptional effects by IFN-β stimulation—both for tissue context-deprived cells and for JAK-STAT pathway mutants. The effect of IFN-β stimulation was most pronounced for core ISGs, but also regulated many other genes that are not classical immune response genes. This broader role for JAK-STAT signaling, which includes genes involved in proliferation and cell cycle, may explain the oncogenic properties of the STAT5B^N642H^ hyperactivation mutant. Our data suggest that this oncogenic driver of T cell leukemia/lymphoma should be thought of as a de novo transcription factor with a set of cancer-associated target genes that is qualitatively different from wild-type STAT5B.

In conclusion, this large-scale analysis of JAK-STAT regulatory programs uncovered diverse roles of JAK-STAT signaling in maintaining immune cell homeostasis. Our results highlight that immune signaling pathways should not be seen as binary on–off switches solely triggered by pathogens and proinflammatory stimuli, but often maintain baseline activity in their in vivo tissue context, with widespread epigenetic and transcriptional implications that help maintain the cells’ regulatory state and their readiness to respond rapidly to immune stimuli. Given that mutations in JAK-STAT pathway members cause diseases such as inborn errors of immunity, inflammatory disorders and cancer^12,13,65,69–73^, it will be interesting to investigate the potential roles of perturbed baseline JAK-STAT signaling in the pathophysiology, diagnosis and treatment of these diseases.

## Methods

### Mouse models

Twelve JAK-STAT mouse models were included in this study: Stat1-ko (*Stat1*^*−/−*^; B6.129P2-*Stat1*^tm1Dlv^)^74^, Stat2-ko (*Stat2*^*−/−*^; B6.129-Stat2^tm1Shnd^)^75^, Stat3-ko (*Stat3*^*fl*^*Vav*^*iCre*^; B6.129-*Stat3*^tm1Vpo^Tg(*vav1-iCre*)^A2Kio^/J)^76,77^, Stat4-ko (*Stat4*^*−/−*^; C57BL/6J-*Stat4*^em3Adpmc^/J; JAX stock no. 028526), Stat5-ko (*Stat5*^*fl*^*Vav*^*iCre*^; B6.129S6-*Stat5b*^tm1Mam^ Stat5a^tm2Mam^/Mmjax(*vav1-iCre*)^A2Kio^/J)^34,77^, Stat6-ko (*Stat6*^*−/−*^; B6.129S2(C)-*Stat6*^tm1Gru^/J, JAX stock no. 005977)^78^, Irf9-ko (*Irf9*^*−/−*^; B6.Cg-*Irf9*^tm1Ttg^)^79^, Tyk2-ko (*Tyk2*^*fl*^*CMV*^Cre^; B6.129P2-*Tyk2*^tm1Biat^Tg(CMV-cre)1Cgn)^80,81^, Stat1a-only (*Stat1*^*α/α*^; B6.129P2-*Stat1beta*^tm1Biat^)^82^, Stat1b-only (*Stat1*^*β/β*^; B6.129P2-*Stat1alpha*^tm1Biat^)^82^, Stat5-hyp (*Stat5b*^*N642H*^; B6N-Tg(*Stat5b*^*N642H*^)^726B*iat*^)^48^ and Tyk2-inact (*Tyk2*^*K293E*^;B6.129P2-Tyk2^tm3.1(K923E)Biat^)^40^. All mouse models were on a C57BL/6N genetic background, with the exception of Stat4-ko, which was on a C57BL/6J background. Mice were kept in specific-pathogen-free conditions according to Federation of European Laboratory Animal Science Associations (FELASA) guidelines, with standard chow diet and water ad libitum. The room temperature for the mice was 20 °C to 22 °C, with relative humidity of 55 ± 10% and 12-h light/dark cycles (light period from 6:00 to 18:00). No in vivo experimental perturbations such as infection or other immune stimuli were used in this study. We refer to this setup as ‘homeostatic conditions’ while acknowledging inevitable variation in the conditions across different animal houses. Mice were bred as approved by the Ethics and Animal Welfare Committee of the University of Veterinary Medicine Vienna in accordance with the university’s guidelines for Good Scientific Practice and authorized by the Austrian Federal Ministry of Education, Science and Research (BMWFW-68.205/0068-WF/V/3b/2015, BMBWF_GZ:2020-0.200.397, BMWFW-68.205/0093-WF/V/3b/2015, BMBWF-68.205/0091-V/3b/2019, BMWFW-68.205/0166-WF/V/3b/2015) in accordance with current legislation. All experiments were performed on cells collected from female mice within an age range of 8–12 weeks.

### Immune cell isolation and purification

We established and validated a standard immune cell isolation and sorting workflow, which was applied consistently across all experiments. Spleens were resected and immediately placed into tubes containing cold PBS (Gibco). Tissue was smashed with a 100-µm strainer (SPL Life Sciences) using a syringe plunger and a 50-ml tube. A new strainer was used for each spleen and rinsed with 10 to 20 ml of DMEM (Gibco) containing 10% FCS (Sigma) and 5 ml of penicillin streptomycin with 10,000 U ml^−1^ (Gibco). For the isolation of dendritic cells, spleens were injected with and placed in a digestion mixture (RPMI (Sigma), 2% FBS, 1 mg ml^−1^ Collagenase D, 20 µg ml^−1^ DNase I) and then incubated at 37 °C for 30 min in a 24-well cell culture dish, before proceeding with the same mashing through a 100-µm strainer. We pooled cells from three littermates to obtain sufficient cell numbers. Samples were centrifuged at 500*g* for 5 min at 4 °C. Pellets were resuspended in 1 ml of Red Blood Cell Lysis Solution (Promega, Z3141) and incubated for 5 min on ice. The lysis was stopped by adding 50 ml of 1 × PBS. Samples were centrifuged at 500*g* for 5 min at 4 °C. Supernatant was discarded and pellets were resuspended in 1 ml of PBS supplemented with 2% BSA (Sigma). Samples were filtered through a 70-µm strainer (SPL Life Sciences). The strainer was washed with 1 ml of PBS supplemented with 2% BSA. MHCII^+^ CD11c^+^ dendritic cells were enriched by magnetic activated cell sorting (MACS) using the Miltenyi Pan Dendritic Cell Isolation Kit (mouse) according to the manufacturer’s instructions (Miltenyi Biotec, 130-100-875). Samples were centrifuged at 500*g* for 5 min at 4 °C and supernatant was discarded.

Cell pellets were resuspended in 100 µl of PBS (2% BSA) and anti-CD16/CD32 (clone 93, Biolegend) was added at a concentration of 1:500 for 15 min to prevent nonspecific binding. Cell suspensions were then stained with combinations of antibodies (all from Biolegend) against TER-119 (APC-Cy7, clone TER-119), F4/80 (FITC, clone BM8), CD19 (PerCP-Cy5.5, clone 6D5), NK1.1 (PE-Cy7, clone PK136, when no NK cells were purified) and CD45 (AF700, clone 30-F11) in a concentration of 1:100; CD8 (APC, clone 53-6.7), CD3 (PE, clone 17A2), Ly-6C (PE-Cy7, clone HK1.4), Ly-6G (PE-Cy7, clone 1A8), NK1.1 (PE-Cy5, clone S17016D, when NK cells were purified) in a concentration of 1:200, and Fixable Viability Dye eFluor 780 (APC-eFluor 780, eBioscience). For dendritic cell purification, we used CD11c (PE-Cy7, clone N418, eBioscience) and MHCII (PE, MHC Class II (I-A/I-E) Monoclonal Antibody (M5/114.15.2), eBioscience) in a concentration of 1:200 and Fixable Viability Dye eFluor 780 (APC-eFluor 780, eBioscience). Cells were stained for 30 min at 4 °C in the dark. Then, 1 ml of PBS supplemented with 2% BSA was added, and suspensions were centrifuged at 500*g* for 5 min at 4 °C. Pellets were resuspended in 300 µl of PBS supplemented with 2% BSA and filtered over a 40-µm strainer (SPL Life Sciences), and filters were rinsed with 1 ml of PBS supplemented with 2% BSA. Cells were sorted with a BD FACS-Aria III Fusion instrument into PBS supplemented with 20% BSA using the gating strategy depicted in Supplementary Fig. 1. Data analysis was performed with the FlowJo v.10 (Tree Star) software. Aliquots of the sort-purified cell populations were stored for RNA/DNA isolation in RLT buffer (Qiagen) or directly processed with the ATAC-seq assay. Due to massive expansion of the T cell compartment in the STAT5B^N642H^ mutant, we were not able to sort-purify sufficient numbers of macrophages from the spleens in a time frame that was compatible with the sort duration for the other genotypes.

### Ex vivo immune cell culture and stimulation

Splenic macrophages and CD8^+^ T cells were cultured in 48-well tissue culture plates for in vitro treatment with the different stimuli. To that end, the cells were centrifuged at 500*g* for 5 min at 4 °C. Then, 1 × 10^5^ macrophages and 3 × 10^5^ T cells were seeded per well in 300 µl of media. Macrophages were resuspended in DMEM (10% FCS, 5 ml of penicillin streptomycin with 10,000 U ml^−1^) and T cells in RPMI (10% FCS, 5 ml of penicillin streptomycin with 10,000 U ml^−1^). The following conditions were applied: (1) 20 h in culture untreated; (2) 20 h in culture with treatment; and (3) 18.5 h in culture followed by treatment for the last 1.5 h. Treatments included murine recombinant IFN-β carrier-free (PBL Assay Science, catalog no. 12401-1) at a final concentration of 1,000 U ml^−1^ or recombinant murine IL-2 (PeproTech, catalog no. 212-12) at a final concentration of 1,000 ng ml^−1^ or murine M-CSF (PeproTech, catalog no. 315-02) at a final concentration of 100 ng ml^−1^.

T cells were collected by transferring them into a reaction tube, adding cold PBS (0.2% BSA), centrifuging at 500*g* for 5 min at 4 °C and removing supernatant. T cells were resuspended in 1 ml of PBS (0.2% BSA) and split equally between the two tubes. Macrophages were collected by removing the supernatant and gently rinsing the cells with cold PBS (0.2% BSA), followed by the addition of cold PBS (0.2% BSA). Macrophages were scraped and equally split between the two tubes. Tubes were then centrifuged at 500*g* for 5 min at 4 °C and either taken for RNA/DNA isolation or ATAC-seq. After centrifugation, the supernatant was carefully removed, and the pellet was resuspended in 350 μl of RLT buffer (Qiagen) with 3.5 µl of β-mercaptoethanol (Sigma). After vortexing the sample for 1 min, it was stored at −80 °C until further processing. RNA and DNA were isolated with the AllPrep RNA/DNA Micro Kit (Qiagen) following the manufacturer’s instructions and stored as recommended.

### Transcriptome profiling with Smart-seq2

We used 500 pg of RNA as input. Reverse transcription and PCR were performed as described^83^. Library preparation was conducted on 1 ng of complementary DNA using the Nextera XT DNA Sample Preparation Kit (Illumina) followed by SPRI (Beckman Coulter) size selection. Sequencing was performed by the Biomedical Sequencing Facility at CeMM using the Illumina HiSeq 3000/4000 platform and the 50-base pair (bp) single-end configuration. Sequencing statistics are provided in Supplementary Table 1.

### Spatial transcriptomics

We used 8–12-week-old mice from either wild-type or STAT1 knockout strains for organ isolation. Mice were euthanized according to institutional guidelines, within an enclosed fume hood. Immediately after euthanasia, the thoracic cavity was opened and a 25 G needle attached to a canula and syringe containing 7.5% formaldehyde was inserted into the ascending aorta within the left ventricle. The needle was secured in position with hemostatic forceps. The right atrium was incised using a pair of fine scissors. Before perfusion, the abdominal cavity was opened to visualize the liver. Perfusion with formaldehyde was performed at an average rate of 5 ml min^−1^, with a total of 20–25 ml of formaldehyde used per animal. Successful perfusion was determined by general stiffness within tissues and pale appearance of the liver. The spleen was dissected carefully with minimal contact and utilizing the fascia associated with the splenic capsule to gently isolate the tissue. The splenic tissue was cut (2 mm) at either end and incubated in formaldehyde for further fixation. Wherever possible, minimal pressure and handling of tissue was employed to avoid disrupting the tissue architecture.

After fixation, tissue specimens were processed using a vacuum infiltration processor (Sakura Tissue-Tek VIP 6 AI) equipped with a graded series of alcohol solutions, xylene and molten paraffin. Subsequently, formalin-fixed samples were embedded into paraffin blocks on an embedding workstation (Thermo Scientific HistoStar) before sectioning on a rotary microtome (Thermo Scientific HM 355S). Before starting, all surfaces and work areas were wiped with ethanol. After trimming excess paraffin, the formalin-fixed paraffin-embedded (FFPE) blocks were placed in an ice bath and incubated for 15 min, before taking 5-µm tissue sections, which were placed on the capture area of a Visium Spatial Gene Expression for FFPE slide (10X Genomics). The slide was placed in a drying rack and incubated in an oven at 42 °C for 3 h, and then placed in a desiccator overnight at room temperature. Subsequent steps to obtain sequencing-ready libraries were performed following the manufacturer’s instructions. Sequencing was performed by the Biomedical Sequencing Facility at CeMM using the Illumina NovaSeq 6000 platform and the 50-bp paired-end configuration on a NovaSeq SP flowcell. Raw sequencing data were processed using the SpaceRanger pipeline v.2.0.0 (10X Genomics) with default parameters. Processed data were analyzed using LoupeCellBrowser v.6.0 (10X Genomics).

### Single-molecule RNA-FISH

Spleens were fixed in 10% formalin for 24 h at room temperature and embedded in paraffin. In situ RNA hybridization was performed using the RNAscope Multiplex Fluorescent Detection Kit v2 (Advanced Cell Diagnostics) with the following target probes: Mm-Oas3 (catalog no. 1054261-C2), Mm-Ifit3 (catalog no. 508251-C2), Mm-Cd3e (catalog no. 314721-C3), using a previously described protocol^84^. After the final amplification step, hybridized probes were visualized using Cy3 or Opal650 conjugated tyramide (Perkin Elmer). Sections incubated with a negative control probe targeting the DapB gene from *Bacillus subtilis* were analyzed in parallel. Positive control probes against murine Ppib and Ubc were used to confirm RNA integrity in each detection channel for each of the analyzed spleens. Images were acquired with a NIKON Eclipse Ti2-E/Yokogawa CSU-W1 confocal spinning disk microscope with a CFI PlanApo λ ×20 objective/0.75 numerical aperture/1 mm working distance and a 50-µm pinhole disc.

### Epigenome profiling with ATAC-seq

Chromatin accessibility mapping by ATAC-seq was performed as previously described^85,86^, with minor adaptations. After centrifugation, the pellet was carefully resuspended in the transposase reaction mix (12.5 µl of 2 × TD buffer, 2 µl of TDE1 (Illumina), 10.25 µl of nuclease-free water and 0.125 µl of 10% NP-40 (Sigma) for macrophages and dendritic cells or 0.25 µl of 1% digitonin (Promega) for all other cell types) and incubated for 30 min at 37 °C. Following DNA purification using the MinElute kit, DNA was eluted in 11 µl. We used 1 µl of the eluted DNA in a quantitative PCR reaction to estimate the optimum number of amplification cycles. The remaining 10 μl of each library was amplified for the number of cycles corresponding to the Cq value from the quantitative PCR (that is, the cycle number at which fluorescence has increased above background levels, rounded down). Library amplification was followed by SPRI bead (Beckman Coulter) size selection to exclude fragments larger than 1,200 bp. DNA concentration was measured with a Qubit fluorometer (Life Technologies). Library amplification was performed using custom Nextera primers^85^. The libraries were sequenced by the Biomedical Sequencing Facility at CeMM using the Illumina HiSeq 3000/4000 platform and the 50-bp single-end configuration. Sequencing statistics are provided in Supplementary Table 1.

### Processing and quality control of the RNA-seq data

RNA-seq data were processed and quality-controlled using established bioinformatics software. Raw reads were trimmed using trimmomatic (v.0.32)^87^ and aligned to the mouse reference genome (mm10) using STAR (v.2.7.1)^88^. Gene expression was quantified by counting uniquely aligned reads in exons using the function summarizeOverlaps from the GenomicAlignments package (v.1.6.3) in R (v.3.2.3). Gene annotations were based on the Ensembl GENCODE Basic set (genome build GRCm38 release 93)^89^. In a first quality control step, samples were excluded that had fewer than 10^6^ reads, an alignment rate below 0.5 or an exome alignment rate below 0.3. Next, outliers were removed based on similarity across biological replicates (that is, samples of the same JAK-STAT mutant, cell type and treatment). To that end, the Spearman correlation between each sample and its replicates was calculated, and samples with a mean correlation below the following cutoffs were excluded as outliers. For homeostatic immune cells, the average Spearman correlation between wild-type macrophage and wild-type CD8^+^ T cells (that is, two clearly distinct and distinguishable cell types) was used as the cutoff. For cultured samples, an arbitrary threshold of 0.5 was used because of the strong effects of cell culture on macrophages (Extended Data Fig. 9a,b). When fewer than three samples passed the cutoff for a given condition, the three samples with highest correlations with each other were kept.

### Processing and quality control of the ATAC-seq data

ATAC-seq data were processed and quality-controlled using established bioinformatics software. Raw reads were trimmed with trimmomatic (v.0.32)^87^ and aligned to the mouse reference genome (mm10) using bowtie2 (v.2.2.4). Primary alignments with mapping quality greater than 30 were retained. ATAC-seq peaks were called using MACS (v.2.7.6)^90^ on each individual sample. Peaks were aggregated into a list of consensus peaks using the function reduce of the package GenomicRanges (v.1.38.0) in R (v.3.6.1). Consensus peaks that overlapped with known blacklisted genomic regions (https://github.com/Boyle-Lab/Blacklist/tree/master/lists) were discarded. Quantitative measurements were obtained by counting reads within consensus peaks using the function summarizeOverlaps from the GenomicAlignments (v.1.22.1) package in R (v.3.6.1). For quality control, samples with fewer than 5 × 10^6^ reads, fewer than 10^3^ peaks, alignment rate lower than 0.5 or fraction of reads overlapping consensus peaks below 0.025 were excluded from the analysis. Moreover, outliers were identified and removed in the same way as for the RNA-seq data, using the average Spearman correlation between wild-type macrophages and wild-type CD8^+^ T cells as cutoff. For the in vitro cultured cells, only untreated cells were used to calculate the cutoff.

### Data analysis software

Data analysis was performed in R (v.3.6.1) using the packages limma (3.42.2)^91^, variancePartition (1.16.1)^92^, edgeR (v.3.28.1)^93^, lme4 (v.1.1.21)^94^, fgsea (v.1.12.0), LOLA (v.1.16.0)^95^, umap (v.0.2.5.0)^96^ and igraph (v.1.2.4.2; https://igraph.org). The HOMER software tool (v.4.11)^97^ was called using Perl (v.5.10.1). Additional enrichment analyses were performed in R (v.4.0.2) using the packages tMOD (v.0.46.2)^98^ and chipenrich (v.2.14.0)^99^. The TOBIAS software (v.0.14.0)^100^ was called using Python (v.3.7.12). Receptor–ligand interaction analysis was performed in R (v.4.2.2) using the packages CellChat (v.1.5.0)^101^ and ProjecTILs (v.3.0.0)^102^.

### Transcriptome analysis of homeostatic immune cells

To dissect the gene-regulatory roles of the different JAK-STAT members, we identified differentially expressed genes using a linear mixed model framework with a fixed categorical effect for the mutants (setting wild type as the baseline reference level) and a random intercept effect for the experiment identifier as nuisance variable, to account for potential batch effects such as the processing date, experimenter, laboratory and genetic background. Hypothesis testing with this model was done using the function dream from the variancePartition package (which is a wrapper for the function lmer from package lme4), separately for each cell type. To obtain maximum likelihood estimates, the option REML was set to false.

Raw read counts were normalized to log_2_ counts per million (log_2_CPM) and gene expression weights were calculated using the function voomWithDreamWeights from the variancePartition package, with normalizing factors calculated using the function calcNormFactors from the edgeR package. Lowly expressed genes with average log_2_CPM below zero were excluded from the analysis. The fitted linear models provided log_2_ fold changes (log_2_FCs) as estimates of effect size and associated *P* values, for each mutant compared with wild type at each tested gene in each cell type. *P* values across all comparisons were adjusted for multiple testing using the false discovery rate (FDR) approach implemented in the function p.adjust in base R with method ‘BH’. Adjusted *P* values (*P*_adj_) lower than 0.05 were declared significant (5% FDR cutoff). For visualization, raw read counts were further normalized to transcripts per million to correct for transcript length.

In addition to the model comparing JAK-STAT mutants with wild type, to test whether differences of genetic backgrounds influence mutant effects, we also performed differential expression analysis as described above but comparing wild-type samples from C57BL/6J mice and C57BL/6N mice.

### Gene set enrichment analyses

To identify enriched biological processes among differentially regulated genes, we performed gene set enrichment analysis for gene sets downloaded from EnrichR^103^, including biological pathways (KEGG_2019_Mouse, NCI-Nature_2016, WikiPathways_2019_Mouse, Reactome_2016), transcription factor target genes (TRANSFAC_and_JASPAR_PWMs, ENCODE_and_ChEA_Consensus_TFs_from_ChIP-X, ENCODE_TF_ChIP-seq_2015, ChEA_2016, TRRUST_Transcription_Factors_2019) and target genes of kinase perturbations (Kinase_Perturbations_from_GEO_down, Kinase_Perturbations_from_GEO_up). In addition, we obtained gene sets related to immune processes including IFN signaling from three sources. First, gene sets were downloaded from MSigDB^104^ (collection 7, ‘immunologic signatures’) and filtered to those relevant to our study by selecting only gene sets with the strings ‘CD8’, ‘IL’, ‘IFN’, ‘MAC’, ‘STAT’ or ‘JAK’ in the name of the gene set. Second, IFN response genes were retrieved from a published analysis of IFN signaling^28^. IFN response genes from 11 cell types were extracted from Supplementary Table 1A of that publication. Genes with a log_2_FC greater than 1 in each cell type were selected as IFN response genes in that cell type. Genes with log_2_FC greater than 1 in all 11 cell types were combined into the ‘ISG core’ signature. Third, data from Interferome.org^105^ were kindly provided by Paul Hertzog and Jamie Gearing, comprising IFN response signatures of multiple individual experiments as well as aggregated core signatures of IFN-α, IFN-β and IFN-γ signaling. Based on these gene sets, enrichment analysis was performed using the function fgsea from the fgsea package and the tmodCERNOtest function from the tmod package. To this end, genes were ranked by the negative log_10_-transformed *P* value of differential expression, multiplied by the sign of the log_2_FC.

### Dimensionality reduction and identification of gene clusters

To visualize similarities and differences in gene expression, we projected strongly differential genes (*P*_adj_ lower than 0.05 and an absolute log_2_FC greater than 2) on two dimensions using the UMAP algorithm. An aggregated matrix of log_2_FC values for mutant and stimulation effects was derived, with genes as rows and coefficients (effects) as columns. This matrix was passed to the umap function from the umap package (with default parameters), which generated a *k*-nearest-neighbor graph and placed all genes in a two-dimensional space based on this graph. We identified gene clusters using graph clustering with random walks on the *k*-nearest-neighbor graph obtained from the UMAP R object. Clustering was performed using the function cluster_walktrap from the igraph package with default parameters. Finally, we performed gene set enrichment analysis on the identified clusters using the function fisher.test in R.

### Epigenome analysis of homeostatic immune cells

To dissect the effect of the different JAK-STAT proteins on the epigenome, we compared ATAC-seq signal intensities between mutant and wild-type mice. Hypothesis testing was performed in analogy to the transcriptome analysis, using a fixed categorical effect for the mutants and a random intercept for the experiment identifier, separately for each cell type. The number of reads in each ATAC-seq consensus region (peak) was normalized to log_2_CPM, and regions with average log_2_CPM below zero were excluded from the analysis. Weights were calculated using the function voomWithDreamWeights from the variancePartition package. To improve computational efficiency, hypothesis testing was done with function lmer from package lme4 directly (this is only a difference in implementation and not in the model itself). *P*_adj_ values lower than 0.05 were declared significant, corresponding to a 5% FDR cutoff.

To interpret epigenome effects of JAK-STAT mutants, we performed a series of enrichment analyses. First, we performed gene set enrichment using the function chipenrich from the chipenrich package^99^ using all supported gene sets for mouse. Second, to identify transcriptional regulators associated with differential regions, we performed motif enrichment analysis using the function findMotifsGenome.pl from HOMER^97^, querying vertebrate motifs with known associated transcription factor; and enrichment of experimentally derived binding sites using the functions runLOLA and cleanLOLA from LOLA^95^, querying all regions defined in the package. Third, to identify transcription factor footprints we used the functions ATACorrect, FootprintScores and BINDetect from TOBIAS^100^, using the JASPAR2022 (ref. ^106^) core nonredundant position frequency matrices from JASPAR. As TOBIAS did not support differential analysis with biological replicates, we performed differential analysis using the dream function from the variancePartition package based on a matrix of footprint mean scores of each transcription factor in each sample, which were obtained from BINDetect and subsequently normalized using the function normalizeQuantiles from the limma package. Finally, aggregate plots were generated using the function PlotAggregate from TOBIAS.

### Analysis of ex vivo cell culture effects

To identify genes and genomic regions affected by context deprivation in cell culture, we compared samples from wild-type mice before and after 20 h of cell culture with short or long IFN-β stimulation, or no stimulation. These analyses were performed separately but analogously for RNA-seq and ATAC-seq data, and hypothesis testing was done separately for each cell type. We used linear fixed-effects models with a fixed categorical effect for culture condition (setting uncultured homeostatic cells as the baseline reference level) and a fixed effect for the experimenter as nuisance variable for this statistically straightforward comparison.

### Analysis of receptor–ligand interactions in vivo

To infer cell–cell interactions of T cells and macrophages in vivo, receptor–ligand interactions were inferred based on single-cell datasets from human and murine spleen cells. Data on human spleen cells were obtained from Tabula Sapiens^107^. Cell types were aggregated into myeloid dendritic cells, CD4^+^ T cells, CD8^+^ T cells, monocytes, NK cells, NK T cells, B cells and plasmacytoid dendritic cells. Data on mouse spleen cells were obtained from Tabula Muris/Tabula Muris Senis^60,108^. T cell subtypes were inferred using ProjecTILs^102^. Based on the above datasets, cellular networks were inferred using the package CellChat^101^.

### Comparison of JAK-STAT mutant and IFN-β stimulation effects

To dissect the effect of JAK-STAT mutants on IFN-β stimulation and vice versa, we compared IFN-β treated with untreated context-deprived cells, and JAK-STAT mutant with wild-type cells. This analysis was restricted to cells maintained in culture. We fitted linear mixed models with three fixed effects: first, a fixed categorical effect of stimulation (‘stimulation effect’) with two factor levels, ‘IFN-β stimulation’ and ‘no stimulation’ (baseline reference level); second, a fixed categorical effect of JAK-STAT mutants (‘mutant effect’), where wild type was used as the baseline reference level; third, an interaction effect between the two previous effects. The stimulation effect thus reflects the effect of IFN-β stimulation compared with untreated cells in wild-type cells, while the mutant effect reflects the effect of JAK-STAT mutants compared with wild type in untreated cells. Interaction effects are present if the change in one factor depends on the other factor. For example, if genes are upregulated upon stimulation in wild type but are not upregulated upon stimulation in the STAT2 knockout, this will lead to a negative interaction effect. The experiment identifier was included as a random effect to account for potential batch effects. Hypothesis testing was performed with the function dream from variancePartition. Data normalization, exclusion of lowly expressed genes and calculation of weights were performed as described above. The fitted models (one per cell type) resulted in three sets of log_2_FC effect size estimates and associated *P* values: one for the stimulation effect, one for the mutant effect and one for the interaction effect between stimulation and mutant effects. To assess statical significance, we applied a 5% FDR cutoff using the function p.adjust in base R with method ‘BH’.

### Grouping of genes based on main and interaction effects

Interaction effects can have different interpretations for different genes, depending on the corresponding main effect. We thus grouped genes with significant interaction effects based on the relative magnitude (log_2_FC) of their main and interaction effects. This grouping was performed separately for each gene, mutant and stimulation. A gene can thus be in different groups for different mutants. The grouping was performed using a multi-step procedure: First, if the absolute log_2_FC of the interaction effect was twofold greater than the absolute log_2_FC of both main effects, the gene was classified as ‘de novo effect’ (group 1). Second, if the absolute log_2_FC of the interaction effect was not twofold greater than the absolute log_2_FC of either main effect, the gene was classified as ‘minor interaction effect’. This group was not further analyzed (no group). Third, if the absolute log_2_FC of the interaction effect was twofold greater than the absolute log_2_FC of the mutation effect but not twofold greater than the absolute log_2_FC of the stimulation effect, then the stimulation effect of the gene was modified by the JAK-STAT mutant. In this case, if the interaction and stimulation effects had the same sign (both positive or both negative), the gene was classified as ‘mutant enhances stimulation effect’ (group 2). If the signs differed, the gene was classified as ‘mutant reverts stimulation effect’ (group 3). Fourth, if the absolute log_2_FC of the interaction effect was twofold greater than the absolute log_2_FC of the stimulation effect but not twofold greater than the absolute log_2_FC of the mutation effect, then the mutant effect of the gene was modified by the stimulation. In this case, if the interaction and mutant effects had the same sign (both positive or both negative), the gene was classified as ‘stimulation enhances mutant effect’ (group 4). If the signs differed, the gene was classified as ‘stimulation reverts mutant effect’ (group 5). To interpret these gene groups, we performed gene set enrichment analyses (as above), using Fisher’s exact test to identify enriched gene sets.

### Reporting summary

Further information on research design is available in the Nature Portfolio Reporting Summary linked to this article.

## Online content

Any methods, additional references, Nature Portfolio reporting summaries, source data, extended data, supplementary information, acknowledgements, peer review information; details of author contributions and competing interests; and statements of data and code availability are available at 10.1038/s41590-024-01804-1.

### Supplementary information

Supplementary Information Supplementary Note, Figs. 1–7 and legends of Supplementary Tables 1–7.

Reporting Summary

Supplementary Tables 1-7 Supplementary Table 1. Sequencing statistics for RNA-seq and ATAC-seq. Supplementary Table 2. Differential gene expression under homeostatic conditions. *P* values were obtained using linear mixed models (two-sided) and corrected for multiple comparisons. Supplementary Table 3. Differential chromatin accessibility under homeostatic conditions. *P* values were obtained using linear mixed models (two-sided) and corrected for multiple comparisons. Supplementary Table 4. Differential gene expression under ex vivo cell culture conditions. *P* values were obtained using linear models (two-sided) and corrected for multiple comparisons. Supplementary Table 5. Differential chromatin accessibility under ex vivo cell culture conditions. *P* values were obtained using linear models (two-sided) and corrected for multiple comparisons. Supplementary Table 6. Receptor–ligand pairs inferred from the Tabula Muris and Tabula Sapiens datasets. *P* values were obtained using random sampling (two-sided test) and corrected for multiple comparisons. Supplementary Table 7. Differential gene expression upon IFN-β stimulation. *P* values were obtained using linear mixed models (two-sided) and corrected for multiple comparisons.

## Data Availability

The Supplementary Website (http://jakstat.bocklab.org) provides data links and genome browser tracks for interactive data visualization. Raw and processed RNA-seq and ATAC-seq data are also available from the NCBI Gene Expression Omnibus (GEO) repository (accession number: GSE204736). Genome assemblies and gene annotations (mm10/GRCm38 release 93) are available from Ensembl (https://ensembl.org).

## Code availability

The source code underlying the presented analyses is available from the Supplementary Website (http://jakstat.bocklab.org) and from the Zenodo repository via 10.5281/zenodo.10649062 (ref. ^109^).
