# Supplementary material for: JAK-STAT signaling maintains homeostasis in T cells and macrophages
Source: Nat Immunol. 2024 Apr 24;25(5):847–59. doi: 10.1038/s41590-024-01804-1 (PMC11065702; doi:10.1038/s41590-024-01804-1)
Supplement: Supplementary file 1 — Supplementary Note, Figs. 1–7 and legends of Supplementary Tables 1–7. [file 41590_2024_1804_MOESM1_ESM.pdf]

nature immunology

Article https://doi.org/10.1038/s41590-024-01804-1

JAK-STAT signaling maintains homeostasis in T cells and macrophages

In the format provided by the authors and unedited

Table of Contents

Supplementary Note 1
Supplementary Figures 4
Legends of Supplementary Tables 11
References 12

Supplementary Note

Additional Results 1: Transcription regulation by JAK-STAT proteins in homeostatic immune cells

We determined JAK-STAT mutant-specific changes in the transcriptome and epigenome (**Fig. 1**) using linear mixed models (mutants were modeled as fixed effects and experimental batches as random effects), which statistically controls for potential biases and batch effects (**Supplementary Fig. 2a**). This analytical approach is particularly suited for our integrative analysis as it allows us to perform each comparison (e.g., between a specific JAK-STAT mutant and its matched control) within a consistent shared framework. As an independent validation of this statistical approach, we also performed separate pairwise comparisons of each JAK-STAT mutant with the matched control samples, and we obtained high consistency with the integrative analysis for all cell types and all mutants (**Supplementary Fig. 2b**). The observed JAK-STAT mutant-specific changes strongly exceeded any effects caused by technical factors (**Extended Data Fig. 2a**) and were clearly distinct from potential confounders including the genetic background of the mice (**Supplementary Fig. 3**). Moreover, our RNA-seq experiments were sufficiently sensitive to detect the relatively low expression of JAK-STAT genes and of many JAK-STAT target genes under homeostatic conditions (**Supplementary Fig. 4**).

In our analysis of the transcriptome profiles across JAK-STAT mutant mice, we found evidence of cell-type-specific feedback loops under homeostatic conditions. STAT1, STAT2, and IRF9 are known to cross-regulate each other via ISGF3-dependent gene regulation[1, 2, 3, 4], and we indeed observed downregulation of *Stat2* and *Irf9* expression in STAT1-deficient macrophages (**Extended Data Fig. 2b**). In contrast, this feedback loop appears to act differently in T cells, where STAT1-deficient mice retained their *Stat2* expression and showed moderately reduced *Irf9* expression. Furthermore, *Stat1* expression was affected by IRF9 knockout but not by STAT2 knockout, and STAT3 deficiency led to an increase in *Stat1* expression in macrophages. These observations are indicative of complex gene-regulatory mechanisms under homeostatic conditions. In contrast, they are not compatible with the hypothesis of a simple ISGF3-dependent binary switch of ISG expression.

We also observed transcriptional changes that were shared by several of the six identified gene clusters (**Figure 1d, Extended Data Fig. 2e; Supplementary Table 2**). For example, TYK2 knockouts in T cells and NK cells mimicked the transcriptional response in STAT2 and IRF9 knockouts, whereas TYK2-deficient macrophages resembled STAT1-deficient macrophages. STAT1 and STAT4 knockouts showed loosely similar effects, consistent with their similar binding specificity and ability to form joint regulatory complexes[5, 6, 7]. Finally, we found that STAT6-dependent genes in macrophages significantly overlapped with STAT5-dependent genes in B cells, indicative of a context-specific division of labor between STAT5 and STAT6.

Additional Results 2: JAK-STAT protein isoforms, mutations, and regulatory processes in homeostasis

In addition to our analysis of STAT2 and IRF9 knockouts (**Fig. 3a**) and of STAT5 knockout and STAT5B^{N642H} (**Fig. 3b**) that are summarized in the main paper, we also investigated the two STAT1 splicing isoforms (**Fig. 3c**) and the kinase-independent effects of TYK2 (**Fig. 3d**), as summarized below.

Knockouts of either the STAT1 alpha or beta isoform had similar effects as the full STAT1 knockout in T cells (Spearman's r = 0.703 for Stat1a-only; Spearman's r = 0.748 for Stat1b-only) but not in macrophages (Spearman's r = 0.176 for Stat1a-only; Spearman's r = 0.184 for Stat1b-only) (**Fig. 3c, top left panel**). This suggests that the two STAT1 isoforms cooperate much more closely in T cells than in macrophages. Nevertheless, both

in T cells and in macrophages, we found that genes strongly downregulated in the full STAT1 knockout were often upregulated when only STAT1 alpha was present but downregulated when only STAT1 beta was present (**Fig. 3c, top left panel**, highlighted by red boxes). Focusing on macrophages, we grouped the STAT1 isoform-dependent genes into those that were shared between STAT1 and STAT2 knockouts (indicative of ISRE-driven genes) and those that were specific to STAT1 knockouts (indicative of GAS-driven genes). ISRE-driven genes were more highly expressed in the presence of STAT1 alpha only compared to STAT1 beta only, suggesting that STAT1 alpha is the preferred isoform for the ISGF3 complex in homeostatic macrophages (**Fig. 3c, top right panels; bottom panel; Extended Data Fig. 4**). A similar pattern was observed for a subset of GAS-driven genes, whereas another subset could not be rescued by either of the two isoforms alone, indicating gene-specific cooperativity of STAT1 isoforms (**Fig. 3c, top right panels; bottom panel; Extended Data Fig. 4)**. Interestingly, cooperativity seems more important for gene repression than for gene activation, as those genes that showed increased expression in the absence of STAT1 (but not STAT2) were frequently upregulated in both STAT1 isoform knockouts (**Fig. 3c, top right panels; bottom panel; Extended Data Fig. 4)**.

Finally, we identified and characterized kinase-independent effects of TYK2 by comparing the transcriptomes of the kinase-dead mutant with the full knockout across four cell types. We observed highly cell type-specific patterns (Spearman's r = 0.740 in macrophages; 0.511 in dendritic cells; 0.480 in NK cells; 0.333 in T cells) (**Fig. 3d, left panel**). In T cells, we found genes related to IL-12 response[8] upregulated in the kinase-dead mutant compared to wildtype, which was not observed in the TYK2 knockout (**Fig. 3d, right panel**). This suggests an important role of kinase-independent TYK2 regulation of IL-12 responsive genes under homeostatic conditions. Other genes that appear to be regulated by kinase-independent effects of TYK2 in T cells include: *Fhod1*, which is involved in the formation of stress fibers in response to infections[9]; *Rtel1* and *Gadd45g*, which are regulators of growth arrest after DNA damage[10, 11], and *Zfp318* with its role B cell function[12] (**Fig. 3d, bottom panel**). Expression of these genes depended on STAT2 and IRF9 but not on STAT1 under homeostatic conditions (**Supplementary Table 2**), suggesting that the regulatory function of IRF9/STAT2 complexes requires TYK2 expression but is independent of TYK2's kinase activity.

Additional Results 3: Chromatin regulation by JAK-STAT proteins in homeostatic immune cells

The chromatin accessibility maps across twelve JAK-STAT mutant mouse models identified a wide range of mutant-specific changes to the epigenome of homeostatic immune cells (**Fig. 5**). The STAT2 and STAT5 knockouts showed the most pronounced loss of chromatin accessibility in the promoter region of their corresponding protein-encoding gene, indicative of strong positive feedback loops (**Extended Data Fig. 6a-c**). Chromatin accessibility at the promoters of classical ISGs depended on the ISGF3 complex members STAT1, STAT2, and IRF9, and on the TYK2 kinase (**Extended Data Fig. 6d**). However, neither of the STAT1 isoform mutants altered the chromatin accessibility of ISG promoters (**Extended Data Fig. 6d**), suggesting redundancy between the two isoforms in terms of their chromatin-regulatory roles. We also observed effects of STAT1 that were not shared by STAT2 and IRF9. For example, knockouts of STAT1, either of its two isoforms, and of TYK2 each resulted in reduced chromatin accessibility at the promoter of the AP-1 family member *Jund* in macrophages. Finally, the hyperactivated $STAT5B^{N642H}$ mutant resulted in strongly increased chromatin accessibility at the *Gata1* gene promoter in T cells (**Extended Data Fig. 6e**), while the STAT5 knockout exhibited increased chromatin accessibility at the *Runx2* and *Trp63* promoters.

Based on changes in the epigenome and transcriptome, we identified three groups of JAK-STAT mutants (**Fig. 5d, bottom panel**). In the first group (characterized by relatively few changes in both the epigenome and transcriptome), STAT1 alpha-only cells (which lack STAT1 beta) showed reduced gene expression and promoter accessibility for the developmental regulator *Setbp1*[13] whereas the cell cycle regulator *E2f7*[14] was affected in STAT1 beta-only cells (which lack STAT1 alpha); STAT4 knockout reduced gene expression and promoter accessibility of the proto-oncogenic transcription factor *Myb* (which has an essential role in the regulation of hematopoiesis[15]) and the proto-oncogene *Met*[16], supporting a role of STAT4 in hematopoietic development; and STAT6 knockout in T cells affected *Dapk3* (which has a pro-apoptotic function[17]) and *Mlycd* (which links STAT6 to fatty acid oxidation as a key metabolic process[18]) (**Extended Data Fig. 7**).

In the second group (characterized by many changes in the transcriptome but relatively few in the epigenome), we detected STAT3-dependent expression of the *Fos* gene in T cells, and of the protein tyrosine phosphatase *Ptprm* as well as the inflammation-associated interleukin *Il1a* in macrophages. STAT3 knockout also resulted in upregulation of immune effector genes in T cells, consistent with STAT3's reported role as a negative regulator of transcription[19]. Affected genes included the lectin-like receptor gene *Klre1*, the *Gzmb* gene encoding granzyme B, and the lymphocyte-associated *Tigit* gene. These results suggest a role of STAT3 as a myeloid-specific suppressor of regulators and effectors important for the lymphoid lineage (**Extended Data Fig. 7**).

In the third group (characterized by many changes in the epigenome), STAT5 knockout upregulated immunoglobin receptor *Tigit*, lectin like receptor *Klrg1*, lymphocyte activator protein 3 (*Lag3*), T cell effectors *Gzmb* and *Gzmk*, and chemokines *Ccl3* and *Ccl4*, which are mediators of inflammatory responses (**Fig. 5e**). We also observed increased chromatin accessibility of NFκB targets in STAT1 and STAT6 knockout macrophages but not T cells, suggestive of cell-type-specific cooperativity of STAT1 and STAT6. Finally, STAT6-dependent genes in T cells and macrophages were enriched for binding of polycomb repressive complex 2 (PRC2) proteins including EZH2 and SUZ12, implicating PRC2 as a potential mediator of STAT6's repressive effect (**Extended Data Fig. 8**).

Supplementary Figures

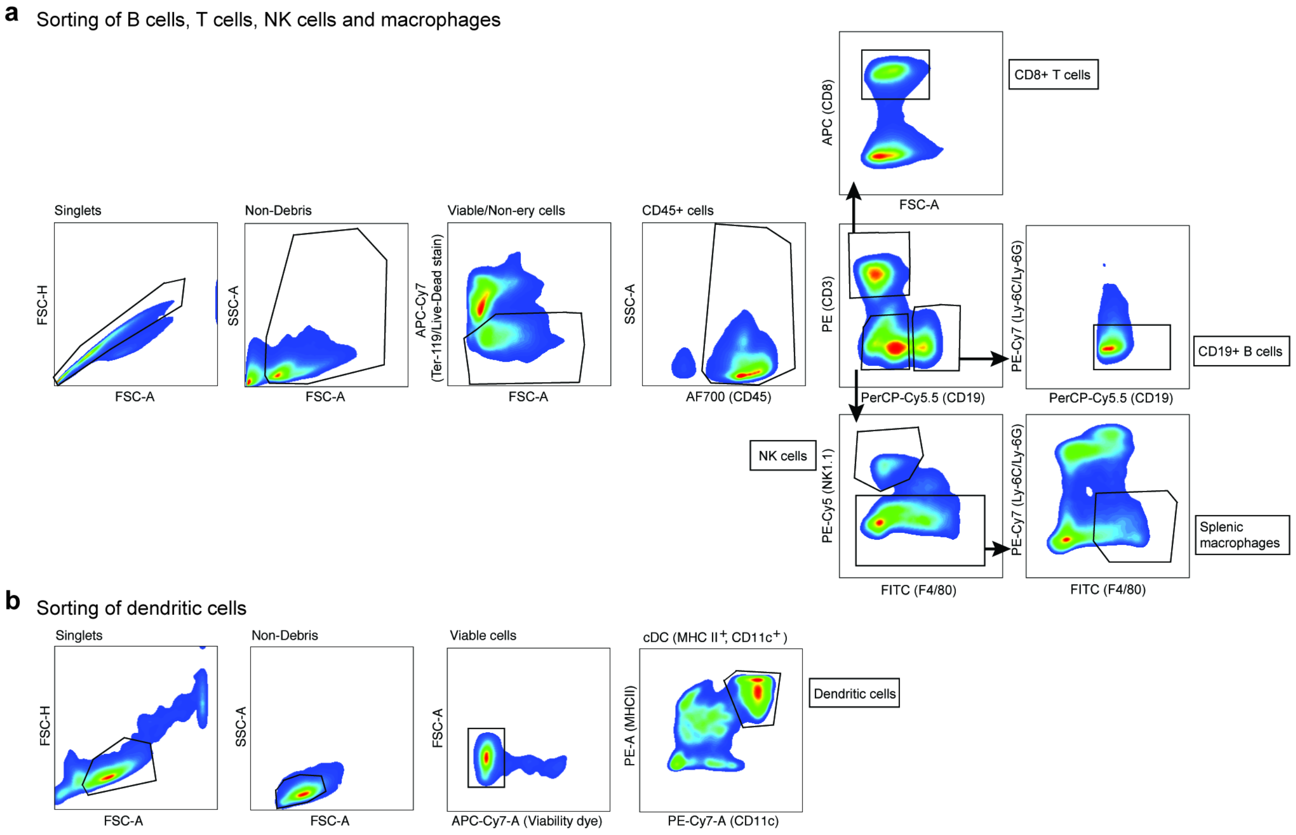

Supplementary Fig. 1 | Illustration of the cell sorting strategy. (**a**) FSC-H and FSC-A was used to determine single cells and SSC-A against FSC-A to define non-debris. Expression of TER-119 (APC-Cy7) was used to exclude erythrocytes. CD45 (AF700) was used to identify immune cells. From this gate we defined T cells as CD3 (PE) positive CD19 (PerCP-Cy5.5) negative, CD8 (APC) positive; B cells as CD19 (PerCP-Cy5.5) positive and Ly-6C/Ly-6G (PE-Cy7) negative; NK cells as CD3 (PE) / CD19 (PerCP-Cy5.5) and F4/80 (FITC) negative, NK1.1 (PE-Cy5) positive. F4/80 positive and Ly-6C/Ly-6G (PE-Cy7) negative cells were taken as macrophages. (**b**) To isolate dendritic cells, we used FSC-H and FSC-A to determine single cells and SSC-A against FSC-A to define non-debris. Viable cells (APC-eFluor 780 negative cells) were selected based on their expression of both CD11c (PE-Cy7) and MHCII (PE).

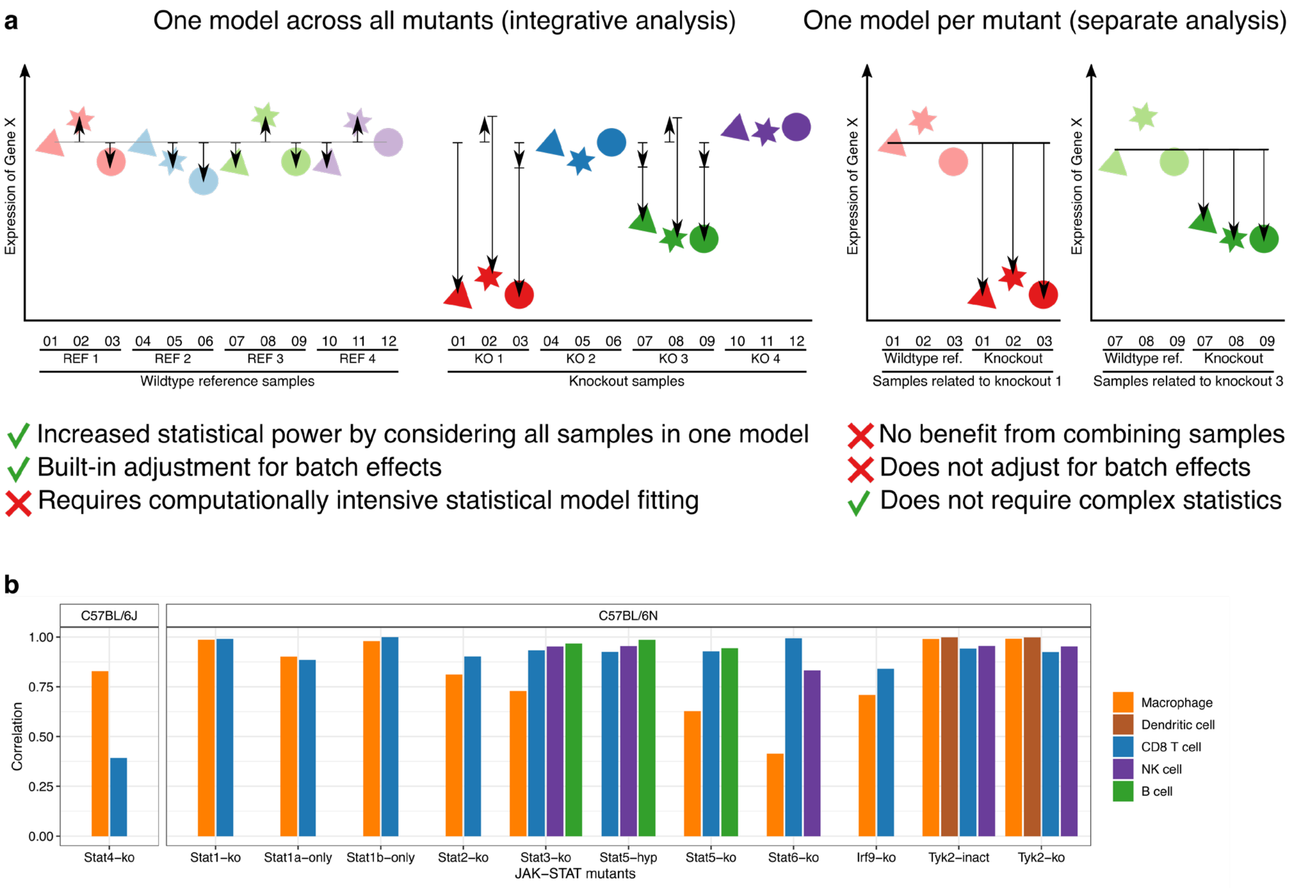

Supplementary Fig. 2 | Validation of the integrative data analysis using an independent statistical approach. (**a**) Outline of the integrative analysis (left), which combines all comparisons in a single linear mixed model with the advantages of increased statistical power and built-in adjustment for batch effects, and of separate analyses (right) with independent pairwise comparisons between each JAK-STAT mutant and its matched experimental control samples. (**b**) Correlation of effect sizes ($\log_2$ fold changes) between both approaches for differentially expressed genes in either analysis. The low-correlation outlier (Stat4-ko in CD8 T cells) is explained by the small number of only 17 differentially expressed genes in this comparison (see also **Fig. 1b**).

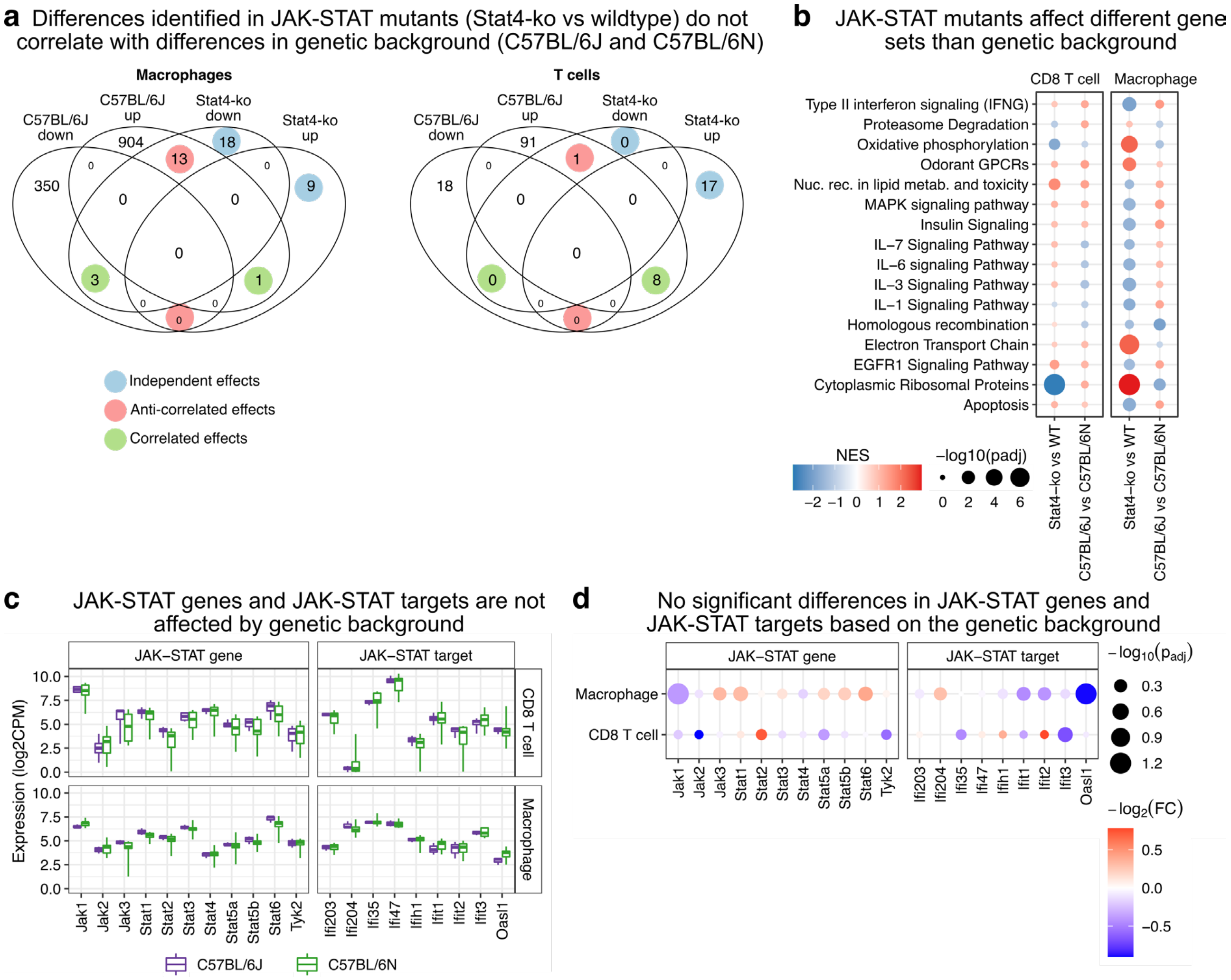

Supplementary Fig. 3 | Limited effects of mouse genetic background on the immune cell transcriptomes. (**a**) Venn diagram of differential genes when comparing genetic background (C57BL/6J versus C57BL/6N) and JAK-STAT mutant to wildtype (STAT4 knockout versus wildtype), for macrophages (left) and T cells (right). Genetic background (mouse strains) did not constitute a major confounder for the observed genotype effects (mutant and wildtype), thus providing further validation of our analytical approach (which compares JAK-STAT mutants to matched wildtype samples from the same experiment and genetic background). (**b**) Gene set enrichment analysis of the differential genes (two-sided random sampling, corrected for multiple comparisons). NES: normalized enrichment scores; padj: adjusted p-value. (**c**) Boxplots showing expression levels for genes involved in JAK-STAT signaling (left) and for JAK-STAT target genes (right), separately for two cell types (top and bottom) and two mouse genetic backgrounds (purple and green). Box plots show the full data range, with the box indicating interquartile range and median. (**d**) Differential expression analysis for the JAK-STAT genes and targets (as in panel c), comparing between the two genetic backgrounds (two-sided linear mixed models, corrected for multiple comparisons). None of the JAK-STAT genes or targets was significantly differentially expressed between the two genetic backgrounds.

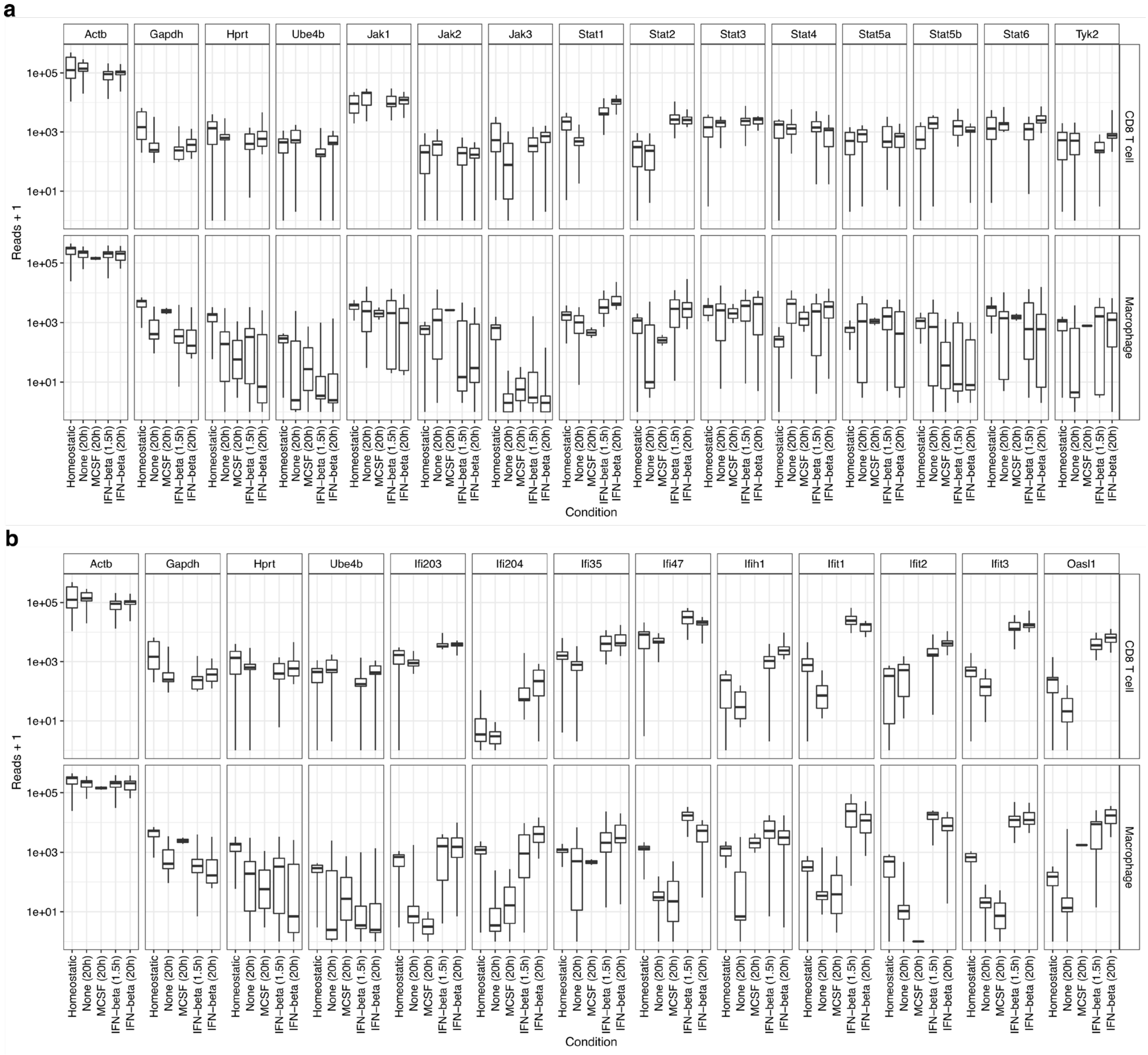

Supplementary Fig. 4 | RNA-seq assay sensitivity for detecting transcription of JAK-STAT genes and targets. Number of RNA-seq reads for genes involved in JAK-STAT signaling (**a**) and for JAK-STAT target genes (**b**). Both gene sets are shown in comparison to reference housekeeping genes (*Actb*, *Gapdh*, *Hprt*, *Ube4b*) in homeostatic *in vivo* samples, as well as cultured samples under different treatments (none, MCSF, IFN-β). Box plots show the full data range, with the box indicating interquartile range and median.

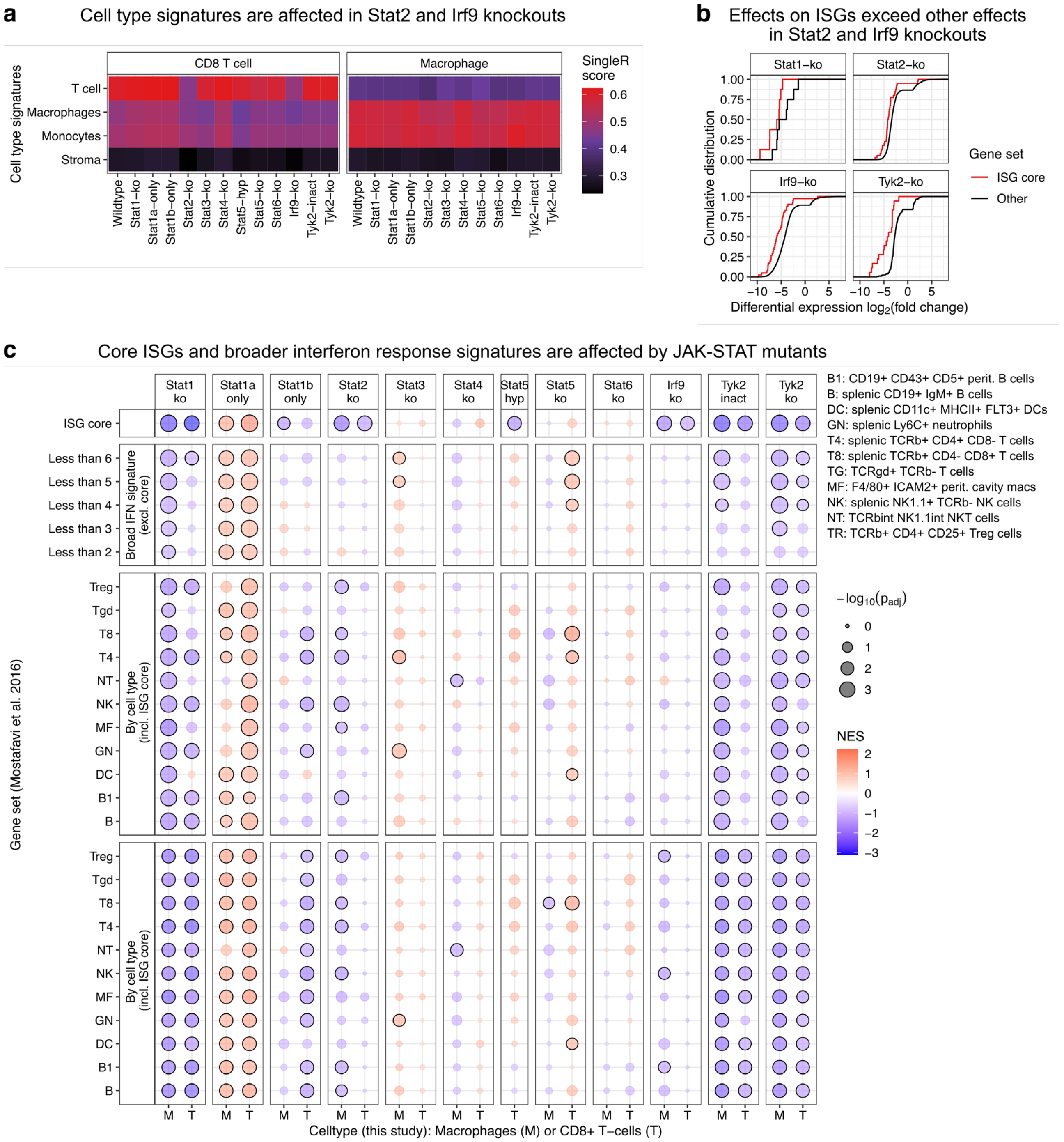

Supplementary Fig. 5 | Comparison of JAK-STAT mutant effects to cell type signatures and broad interferon responses. (**a**) SingleR similarity scores[20] comparing JAK-STAT-mutant and wildtype cells to external reference profiles from the ImmGen consortium[21]. (**b**) Comparison of differential expression of core ISGs to other significant genes. (**c**) Gene set enrichment analysis comparing enrichment of core ISGs to enrichment of broad interferon response signatures across JAK-STAT mutants (two-sided random sampling, corrected for multiple comparisons). All gene sets are based on data from Mostafavi and colleagues[22]. Core ISGs are genes identified in all 11 cell types. Broader interferon response signatures are genes that were significantly regulated in five or fewer cell types (and do not include core ISGs). Cell-type-specific signatures either include core ISGs (all genes significantly regulated per cell type) or exclude them (genes that are found in a maximum of 5 out of 11 cell types). NES: normalized enrichment score; padj: adjusted p-value.

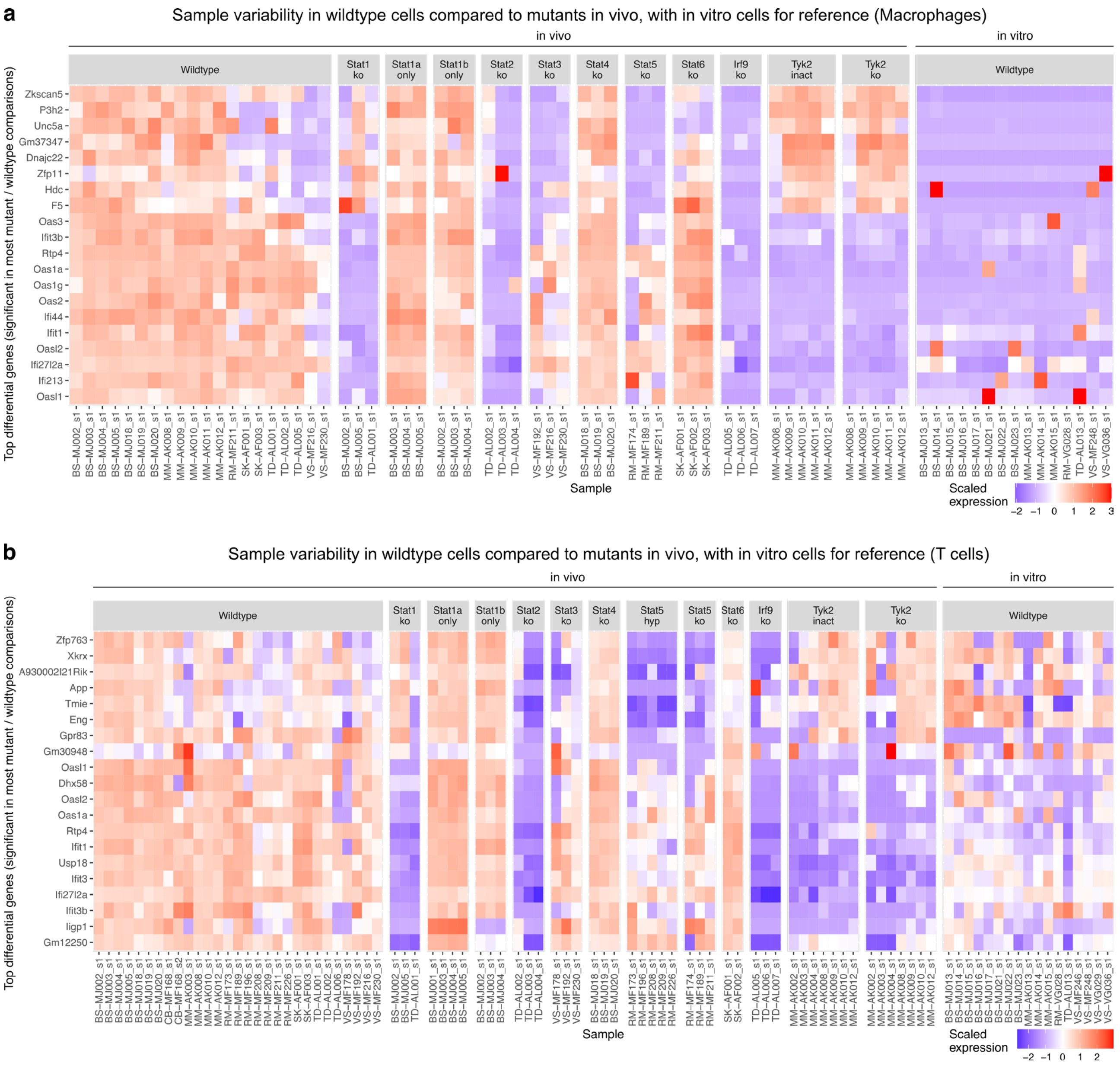

Supplementary Fig. 6 | Sample-to-sample transcriptome variability for wildtype, mutant, and context-deprived cells. Transcriptome variability of wildtype samples is largely exceeded by the effects of JAK-STAT mutants and context deprivation. Expression of selected differential genes in wildtype and mutant samples under homeostatic conditions *in vivo* and in wildtype samples under ex vivo / context-deprivation in macrophages (**a**) and T cells (**b**). Genes with large effects across multiple JAK-STAT mutants are shown. Experiment identifiers are indicated as column labels and capture variability across different mouse houses, genetic backgrounds, and other experimental variables.

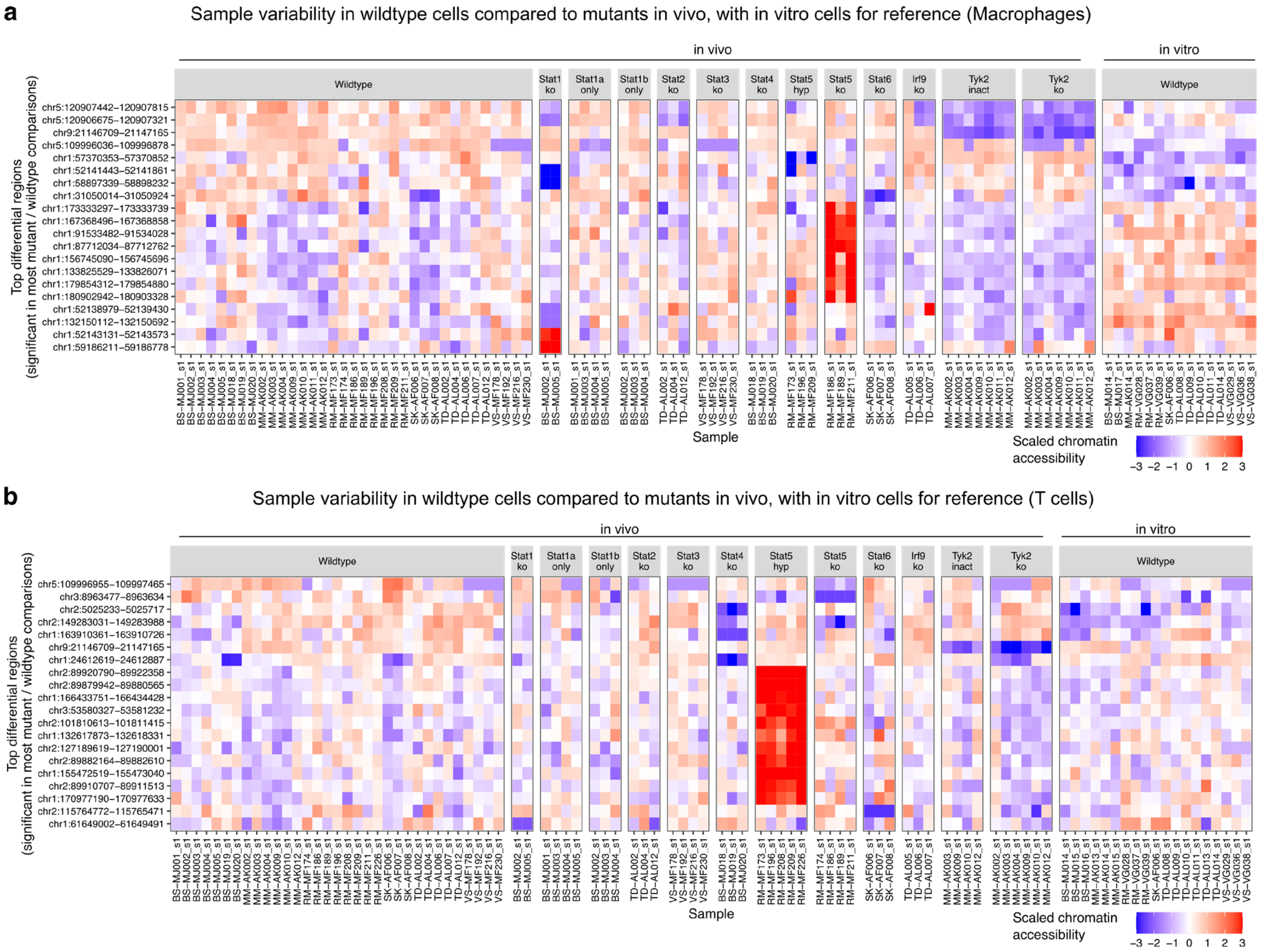

Supplementary Fig. 7 | Sample-to-sample epigenome variability for wildtype, mutant, and context-deprived cells. Chromatin accessibility of selected differential regions in wildtype and mutant samples under homeostasis in vivo and in wildtype samples under ex vivo / context-deprivation in macrophages (**a**) and T cells (**b**). Chromatin accessibility of genomic region with large effects across multiple JAK-STAT mutants is shown. Experiment identifiers are indicated as column labels and capture variability across different mouse houses, genetic backgrounds, and other experimental variables.

Legends of Supplementary Tables

Supplementary Table 1 | Sequencing statistics for RNA-seq and ATAC-seq profiling.

Supplementary Table 2 | Differential gene expression under homeostatic conditions. P-values were obtained using linear mixed models (two-sided) and corrected for multiple comparisons.

Supplementary Table 3 | Differential chromatin accessibility under homeostatic conditions. P-values were obtained using linear mixed models (two-sided) and corrected for multiple comparisons.

Supplementary Table 4 | Differential gene expression under *ex vivo* cell culture conditions. P-values were obtained using linear models (two-sided) and corrected for multiple comparisons.

Supplementary Table 5 | Differential chromatin accessibility under *ex vivo* cell culture conditions. P-values were obtained using linear models (two-sided) and corrected for multiple comparisons.

Supplementary Table 6 | Receptor-ligand pairs inferred from the Tabula Muris and Tabula Sapiens datasets. P-values were obtained using random sampling (two-sided test) and corrected for multiple comparisons.

Supplementary Table 7 | Differential gene expression upon IFN-β stimulation. P-values were obtained using linear mixed models (two-sided) and corrected for multiple comparisons.

References

1. Blaszczyk, K. *et al.* The unique role of STAT2 in constitutive and IFN-induced transcription and antiviral responses. *Cytokine Growth Factor Rev* **29**, 71-81 (2016).
2. Gough, D.J., Messina, N.L., Clarke, C.J., Johnstone, R.W. & Levy, D.E. Constitutive type I interferon modulates homeostatic balance through tonic signaling. *Immunity* **36**, 166-174 (2012).
3. Platanitis, E. *et al.* A molecular switch from STAT2-IRF9 to ISGF3 underlies interferon-induced gene transcription. *Nat Commun* **10**, 2921 (2019).
4. Taniguchi, T. & Takaoka, A. A weak signal for strong responses: interferon-alpha/beta revisited. *Nat Rev Mol Cell Biol* **2**, 378-386 (2001).
5. Collison, L.W. *et al.* The composition and signaling of the IL-35 receptor are unconventional. *Nat Immunol* **13**, 290-299 (2012).
6. Delgoffe, G.M. & Vignali, D.A. STAT heterodimers in immunity: A mixed message or a unique signal? *Jakstat* **2**, e23060 (2013).
7. Yamamoto, K. *et al.* Stat4, a novel gamma interferon activation site-binding protein expressed in early myeloid differentiation. *Mol Cell Biol* **14**, 4342-4349 (1994).
8. Agarwal, P. *et al.* Gene regulation and chromatin remodeling by IL-12 and type I IFN in programming for CD8 T cell effector function and memory. *J Immunol* **183**, 1695-1704 (2009).
9. Schulze, N. *et al.* FHOD1 regulates stress fiber organization by controlling the dynamics of transverse arcs and dorsal fibers. *J Cell Sci* **127**, 1379-1393 (2014).
10. Tamura, R.E. *et al.* GADD45 proteins: central players in tumorigenesis. *Curr Mol Med* **12**, 634-651 (2012).
11. Vannier, J.B., Sarek, G. & Boulton, S.J. RTEL1: functions of a disease-associated helicase. *Trends Cell Biol* **24**, 416-425 (2014).
12. Enders, A. *et al.* Zinc-finger protein ZFP318 is essential for expression of IgD, the alternatively spliced Igh product made by mature B lymphocytes. *Proc Natl Acad Sci U S A* **111**, 4513-4518 (2014).
13. Piazza, R. *et al.* SETBP1 induces transcription of a network of development genes by acting as an epigenetic hub. *Nat Commun* **9**, 2192 (2018).
14. de Bruin, A. *et al.* Identification and characterization of E2F7, a novel mammalian E2F family member capable of blocking cellular proliferation. *J Biol Chem* **278**, 42041-42049 (2003).
15. Soza-Ried, C., Hess, I., Netuschil, N., Schorpp, M. & Boehm, T. Essential role of c-myb in de-finitive hematopoiesis is evolutionarily conserved. *Proc Natl Acad Sci U S A* **107**, 17304-17308 (2010).
16. Tovar, E.A. & Graveel, C.R. MET in human cancer: germline and somatic mutations. *Ann Transl Med* **5**, 205 (2017).
17. Das, T.P. *et al.* Activation of AKT negatively regulates the pro-apoptotic function of death-associated protein kinase 3 (DAPK3) in prostate cancer. *Cancer Lett* **377**, 134-139 (2016).
18. Yizhak, K. *et al.* Phenotype-based cell-specific metabolic modeling reveals metabolic liabilities of cancer. *Elife* **3** (2014).
19. El Kasmi, K.C. *et al.* Cutting edge: A transcriptional repressor and corepressor induced by the STAT3-regulated anti-inflammatory signaling pathway. *J Immunol* **179**, 7215-7219 (2007).
20. Aran, D. *et al.* Reference-based analysis of lung single-cell sequencing reveals a transitional profibrotic macrophage. *Nat Immunol* **20**, 163-172 (2019).
21. Heng, T.S. & Painter, M.W. The Immunological Genome Project: networks of gene expression in immune cells. *Nat Immunol* **9**, 1091-1094 (2008).
22. Mostafavi, S. *et al.* Parsing the Interferon Transcriptional Network and Its Disease Associations. *Cell* **164**, 564-578 (2016).
