# Supplementary material for: JAK-STAT signaling maintains homeostasis in T cells and macrophages
Source: Nat Immunol. 2024 Apr 24;25(5):847–59. doi: 10.1038/s41590-024-01804-1 (PMC11065702; doi:10.1038/s41590-024-01804-1)
Supplement: Supplementary file 2 — Reporting Summary [file 41590_2024_1804_MOESM2_ESM.pdf]

nature portfolio

Corresponding author(s): Christoph Bock, Matthias Farlik

Last updated by author(s): Feb 24, 2024

Reporting Summary

Nature Portfolio wishes to improve the reproducibility of the work that we publish. This form provides structure for consistency and transparency in reporting. For further information on Nature Portfolio policies, see our Editorial Policies and the Editorial Policy Checklist.

Statistics

For all statistical analyses, confirm that the following items are present in the figure legend, table legend, main text, or Methods section.

n/a	Confirmed	
☐	☒	The exact sample size (*n*) for each experimental group/condition, given as a discrete number and unit of measurement
☐	☒	A statement on whether measurements were taken from distinct samples or whether the same sample was measured repeatedly
☐	☒	The statistical test(s) used AND whether they are one- or two-sided<br>*Only common tests should be described solely by name; describe more complex techniques in the Methods section.*
☐	☒	A description of all covariates tested
☐	☒	A description of any assumptions or corrections, such as tests of normality and adjustment for multiple comparisons
☐	☒	A full description of the statistical parameters including central tendency (e.g. means) or other basic estimates (e.g. regression coefficient) AND variation (e.g. standard deviation) or associated estimates of uncertainty (e.g. confidence intervals)
☐	☒	For null hypothesis testing, the test statistic (e.g. *F*, *t*, *r*) with confidence intervals, effect sizes, degrees of freedom and *P* value noted<br>*Give P values as exact values whenever suitable.*
☒	☐	For Bayesian analysis, information on the choice of priors and Markov chain Monte Carlo settings
☐	☒	For hierarchical and complex designs, identification of the appropriate level for tests and full reporting of outcomes
☐	☒	Estimates of effect sizes (e.g. Cohen's *d*, Pearson's *r*), indicating how they were calculated

Our web collection on statistics for biologists contains articles on many of the points above.

Software and code

Policy information about availability of computer code

Data collection	RNA-seq data were processed and quality-controlled using established bioinformatics software. Raw reads were trimmed using trimmomatic (version 0.32) and aligned to the mouse reference genome (mm10) using STAR (version 2.7.1). Gene expression was quantified by counting uniquely aligned reads in exons using the function summarizeOverlaps from the GenomicAlignments package (version 1.6.3) in R (version 3.2.3). Gene annotations were based on the Ensembl GENCODE Basic set (genome build GRCm38 release 93).<br><br>ATAC-seq data were processed and quality-controlled using established bioinformatics software. Raw reads were trimmed with trimmomatic (version 0.32) and aligned to the mouse reference genome (mm10) using bowtie2 (version 2.2.4). Primary alignments with mapping quality greater than 30 were retained. ATAC-seq peaks were called using MACS (version 2.7.6) on each individual sample. Peaks were aggregated into a list of consensus peaks using the function reduce of the package GenomicRanges (version 1.38.0) in R (version 3.6.1). Quantitative measurements were obtained by counting reads within consensus peaks using the function summarizeOverlaps from the package GenomicAlignments (version 1.22.1).
Data analysis	Data analysis was performed in R (version 3.6.1) using the packages limma (3.42.2), variancePartition (1.16.1), edgeR (version 3.28.1), lme4 (version 1.1.21), fgsea (version 1.12.0), LOLA (version 1.16.0), umap (version 0.2.5.0), and igraph (version 1.2.4.2). The HOMER software tool (version 4.11) was called using Perl (version 5.10.1). Additional enrichment analyses were performed in R (version 4.0.2) using the packages tMOD (version 0.46.2) and chipenrich (version 2.14.0). The TOBIAS software (version 0.14.0) was called using Python (version 3.7.12). Receptor-ligand interaction analysis was performed in R (version 4.2.2) using the packages CellChat (version 1.5.0)120 and ProjecTILs (version 3.0.0). The Supplementary Website (http://jakstat.bocklab.org) provides data links and the source code underlying the presented analyses. FACS data analysis was performed with the FlowJo v10.6.1 (Tree Star) software. For spatial transcriptomics raw sequencing data were processed using the SpaceRanger pipeline version 2.0.0 (10X Genomics) with default parameters. Processed data were analyzed using LoupeCellBrowser version 6.0 (10X Genomics).

The source code underlying the presented analyses is available from the Supplementary Website (http://jakstat.bocklab.org) and from the Zenodo repository (https://doi.org/10.5281/zenodo.10649062).

For manuscripts utilizing custom algorithms or software that are central to the research but not yet described in published literature, software must be made available to editors and reviewers. We strongly encourage code deposition in a community repository (e.g. GitHub). See the Nature Portfolio guidelines for submitting code & software for further information.

Data

Policy information about availability of data

All manuscripts must include a data availability statement. This statement should provide the following information, where applicable:

- Accession codes, unique identifiers, or web links for publicly available datasets
- A description of any restrictions on data availability
- For clinical datasets or third party data, please ensure that the statement adheres to our policy

The Supplementary Website (http://jakstat.bocklab.org) provides data links and genome browser tracks for interactive data visualization. Raw and processed RNA-seq and ATAC-seq data are also available from the NCBI Gene Expression Omnibus (GEO) repository (accession number: GSE204736). Genome assemblies and gene annotations (mm10 / GRCm38 release 93) are available from Ensembl (https://ensembl.org).

Human research participants

Policy information about studies involving human research participants and Sex and Gender in Research.

Reporting on sex and gender	Not applicable
Population characteristics	Not applicable
Recruitment	Not applicable
Ethics oversight	Not applicable

Note that full information on the approval of the study protocol must also be provided in the manuscript.

Field-specific reporting

Please select the one below that is the best fit for your research. If you are not sure, read the appropriate sections before making your selection.

☒ Life sciences ☐ Behavioural & social sciences ☐ Ecological, evolutionary & environmental sciences

For a reference copy of the document with all sections, see nature.com/documents/nr-reporting-summary-flat.pdf

Life sciences study design

All studies must disclose on these points even when the disclosure is negative.

Sample size	No sample size calculation was performed. Three independent biological replicates were obtained as is common practice in the field.
Data exclusions	Samples with fewer than one million reads, alignment rate below 50%, or exome alignment rate (for RNA-seq) below 30% were excluded. Outliers were removed based on similarity across biological replicates (i.e., samples of the same JAK-STAT mutant, cell type, and treatment). To that end, the Spearman correlation coefficients between each sample and its biological replicates were calculated, and samples with mean correlation below the following cutoffs were excluded as outliers: For homeostatic immune cells, the average Spearman correlation between wildtype macrophage and wildtype CD8+ T cells (two clearly distinct and distinguishable cell types) was used as the cutoff. In cultured samples, an arbitrary threshold of 0.5 was used because of the strong effects of cell culture on macrophages. The three samples with highest correlations were always kept, in order to maintain an adequate representation of conditions with intrinsically variable transcriptomes.
Replication	Sample collection was repeated until three independent biological replicates passed the filtering on data quality (described above).
Randomization	Processing of samples from the various genotypes and cell types was recorded and did not follow any particular order. Experimental batches were included as covariates in mixed linear models.
Blinding	No blinding of genotypes or treatments was implemented in this study. To control for potential biases and batch effects, each experiment included mutant mice together with matched wildtype mice of the same strain that were processed in the same batch.

Reporting for specific materials, systems and methods

We require information from authors about some types of materials, experimental systems and methods used in many studies. Here, indicate whether each material, system or method listed is relevant to your study. If you are not sure if a list item applies to your research, read the appropriate section before selecting a response.

Materials & experimental systems

n/a	Involved in the study
☐	☒ Antibodies
☒	☐ Eukaryotic cell lines
☒	☐ Palaeontology and archaeology
☐	☒ Animals and other organisms
☒	☐ Clinical data
☒	☐ Dual use research of concern

Methods

n/a	Involved in the study
☒	☐ ChIP-seq
☐	☒ Flow cytometry
☒	☐ MRI-based neuroimaging

Antibodies

Antibodies used

Cell pellets were resuspended in 100 µl PBS (2% BSA) and anti-CD16/CD32 (clone 93, Cat #101302, Biolegend) was added at a concentration of 1:500 for 15 min to prevent nonspecific binding. Cell suspensions were then stained with combinations of antibodies (all from Biolegend) against TER-119 (APC-Cy7, clone TER-119, Cat #116223), CD8 (APC, clone 53-6.7, Cat #100712), F4/80 (FITC, clone BM8, Cat #123108), CD19 (PerCP-Cy5.5, clone 6D5, Cat #115534), CD3 (PE, clone 17A2, Cat #100206), Ly-6C (PE-Cy7, clone HK1.4, Cat #128018), Ly-6G (PECy7, clone 1A8, Cat #127618), NK1.1 (PE-Cy7, clone PK136, Cat #108714, if NK cells were not purified) or NK1.1 (PE-Cy5, clone S17016D, Cat #156524, if NK cells were purified), and CD45 (AF700, clone 30-F11, Cat #103128), and Fixable Viability Dye eFluor 780 (APC-eFluor 780, eBioscience, Cat #65-0865-18). For dendritic cell purification, we used CD11c (PE-Cy7, clone N418, eBioscience, Cat #25-0114-82), MHCII (PE, MHC Class II (I-A/I-E) Monoclonal Antibody (M5/114.15.2, eBioscience, Cat #12-5321-82). Cells were stained for 30 min at 4 °C in the dark.

Validation

All antibodies were purchased from commercial vendors as indicated. Each lot of the antibodies purchased from Biolegend or eBioscience was quality controlled by immunofluorescent staining with flow cytometric analysis, according to the manufacturers protocol. Species specificity was assessed by staining of mouse splenocytes or cells from the bone marrow of C57BL/6 mice. FACS profiles of these tests are available from the manufacturers website following the catalog numbers as depicted above.

Animals and other research organisms

Policy information about studies involving animals; ARRIVE guidelines recommended for reporting animal research, and Sex and Gender in Research

Laboratory animals

The following twelve JAK-STAT mice were included in this study: Stat1-ko (Stat1-/-; B6.129P2-Stat1tm1Dlv), Stat2-ko (Stat2-/-; B6.129-Stat2tm1Shnd), Stat3-ko (Stat3flVaviCre; B6.129-Stat3tm1VpoTg(vav1-iCre)A2Kio/J), Stat4-ko (Stat4-/-; C57BL/6J-Stat4em3Adpmc/J JAX stock #028526), Stat5-ko (Stat5flVaviCre; B6.129S6-Stat5btm1Mam Stat5atm2Mam/Mmjax(vav1-iCre)A2Kio/J), Stat6-ko (Stat6-/-; B6.129S2(C)-Stat6tm1Gru/J, JAX stock #005977), Irf9-ko (Irf9-/-; B6.Cg-Irf9tm1Ttg), Tyk2-ko (Tyk2flCMVCre; B6.129P2-Tyk2tm1BiatTg(CMV-cre)1Cgn), Stat1a-only (Stat1α/α; B6.129P2-Stat1betatm1Biat), Stat1b-only (Stat1β/β; B6.129P2-Stat1alphatm1Biat), Stat5-hyp (Stat5bN642H; B6N-Tg(Stat5bN642H)726Biat), and Tyk2-inact (Tyk2K293E;B6.129P2-Tyk2tm3.1(K923E)Biat).

All mouse models were on a C57BL/6N genetic background, with the exception of Stat4-ko, which was on a C57BL/6J background.

Wild animals

The study did not involve wild animals

Reporting on sex

All experiments were performed on cells collected from female mice within an age range of 8 to 12 weeks

Field-collected samples

The study did not contain samples collected from the field

Ethics oversight

Mice were bred at the University of Veterinary Medicine Vienna as approved by the Ethics and Animal Welfare Committee of the University of Veterinary Medicine Vienna in accordance with the university's guidelines for Good Scientific Practice and authorized by the Austrian Federal Ministry of Education, Science and Research (BMWFW-68.205/0068-WF/V/3b/2015, BMBWF_GZ:2020-0.200.397, BMWFW-68.205/0093-WF/V/3b/2015, BMBWF-68.205/0091-V/3b/2019, BMWFW-68.205/0166-WF/V/3b/2015) in accordance with current legislation.

Note that full information on the approval of the study protocol must also be provided in the manuscript.

Flow Cytometry

Plots

Confirm that:

☒ The axis labels state the marker and fluorochrome used (e.g. CD4-FITC).

☒ The axis scales are clearly visible. Include numbers along axes only for bottom left plot of group (a 'group' is an analysis of identical markers).

☒ All plots are contour plots with outliers or pseudocolor plots.

☒ A numerical value for number of cells or percentage (with statistics) is provided.

Methodology

Sample preparation

We established and validated an immune cell isolation and sorting workflow, which was applied consistently across all experiments. Spleens were resected and immediately placed into tubes containing cold PBS (Gibco). Tissue was smashed with a 100 µm strainer (SPL Life Sciences) using a syringe plunger and a 50 ml tube. A new strainer was used for each spleen and rinsed with 10 to 20 ml DMEM (Gibco) containing 10% FCS (Sigma) and 5 ml penicillin streptomycin with 10,000 U/ml (Gibco). For the isolation of dendritic cells (DCs) spleens were injected with and placed in a digestion mixture (RPMI (Sigma), 2% FBS, 1 mg/ml Collagenase D, 20 µg/ml DNase I) and then incubated at 37 °C for 30 minutes in a 24-well cell culture dish, before proceeding with the same mashing through a 100 µm strainer. We pooled cells from three littermates to obtain sufficient cell numbers. Samples were centrifuged at 500 g for 5 min at 4 °C. Pellets were resuspended in 1 ml Red Blood Cell Lysis Solution (Promega, Z3141) and incubated for 5 min on ice. The lysis was stopped by adding 50 ml 1x PBS. Samples were centrifuged at 500 g for 5 min at 4 °C. Supernatant was discarded and pellets were resuspended in 1 ml PBS supplemented with 2% BSA (Sigma). Samples were filtered through a 70 µm strainer (SPL Life Sciences). The strainer was washed with 1 ml PBS supplemented with 2% BSA. MHCII+ CD11c+ dendritic cells were enriched by magnetic activated cell sorting (MACS) using the Miltenyi Pan Dendritic Cell Isolation Kit (mouse) according to the manufacturer's instructions (Miltenyi Biotec, 130-100-875). Samples were centrifuged at 500 g for 5 min at 4 °C and supernatant was discarded.

Cell pellets were resuspended in 100 µl PBS (2% BSA) and anti-CD16/CD32 (clone 93, Biolegend) was added at a concentration of 1:500 for 15 min to prevent nonspecific binding. Cell suspensions were then stained with combinations of antibodies (all from Biolegend) against TER-119 (APC-Cy7, clone TER-119), F4/80 (FITC, clone BM8), CD19 (PerCP-Cy5.5, clone 6D5), NK1.1 (PE-Cy7, clone PK136, if NK cells were not purified) and CD45 (AF700, clone 30-F11) in a concentration of 1:100; CD8 (APC, clone 53-6.7), CD3 (PE, clone 17A2), Ly-6C (PE-Cy7, clone HK1.4), Ly-6G (PECy7, clone 1A8), NK1.1 (PE-Cy5, clone S17016D, if NK cells were purified) in a concentration of 1:200, and Fixable Viability Dye eFluor 780 (APC-eFluor 780, eBioscience). For dendritic cell purification, we used CD11c (PE-Cy7, clone N418, eBioscience), MHCII (PE, MHC Class II (I-A/I-E) Monoclonal An-tibody (M5/114.15.2), eBioscience) in a concentration of 1:200 and Fixable Viability Dye eFluor 780 (APC-eFluor 780, eBioscience). Cells were stained for 30 min at 4 °C in the dark. 1 ml PBS supple-mented with 2% BSA was added and suspensions were centrifuged at 500 g for 5 min at 4 °C. Pellets were resuspended in 300 µl PBS supplemented with 2% BSA and filtered over 40 µm strainer (SPL Life Sciences), filters were rinsed with 1 ml PBS supplemented with 2% BSA. Cells were sorted with a BD FACS-Aria III Fusion instrument into PBS supplemented with 20% BSA using the gating strategy depicted in Supplementary Fig. 1. Data analysis was performed with the FlowJo v10.6.1 (Tree Star) software. Aliquots of the sort-purified cell populations were stored for RNA/DNA isolation in RLT-buffer (Qiagen) or directly processed with the ATAC-seq assay. Due to massive expansion of the T cell compartment in the STAT5BN642H mutant, we were not able to sort-purify sufficient numbers of macrophages from the spleens in a time frame that was compatible with the sort duration for the other genotypes.

Instrument

BD FACS-Aria III Fusion

Software

FlowJo v10.6.1 (Tree Star). No absolute or relative abundances were calculated in this study

Cell population abundance

The FACS purified fraction contained only one sorted population. Purity was assessed by re-sorting an aliquot of the sorted fraction and confirming that more than 98% of all cells were detectable in the gates required to select the population.

Gating strategy

FSC-H and FSC-A was used to determine single cells and SSC-A against FSC-A to define non-debris. Expression of TER-119 (APC-Cy7) was used to exclude erythrocytes. CD45 (AF700) was used to identify immune cells. From this gate we defined T cells as CD3 (PE) positive CD19 (PerCP-Cy5.5) negative, CD8 (APC) positive; B cells as CD19 (PerCP-Cy5.5) positive and Ly-6C/Ly-6G (PE-Cy7) negative; NK cells as CD3 (PE) / CD19 (PerCP-Cy5.5) and F4/80 (FITC) negative, NK1.1 (PE-Cy5) positive. F4/80 positive and Ly-6C/Ly-6G (PE-Cy7) negative cells were taken as macrophages.

To isolate dendritic cells, we used FSC-H and FSC-A to determine single cells and SSC-A against FSC-A to define non-debris. Viable cells (APC-eFluor 780 negative cells) were selected based on their expression of both CD11c (PE-Cy7) and MHCII (PE).

☒ Tick this box to confirm that a figure exemplifying the gating strategy is provided in the Supplementary Information.
